# Glucocorticoids increase tissue cell protection against pore-forming toxins from pathogenic bacteria

**DOI:** 10.1038/s42003-023-04568-w

**Published:** 2023-02-17

**Authors:** Thomas J. R. Ormsby, Sian E. Owens, Matthew L. Turner, James G. Cronin, John J. Bromfield, I. Martin Sheldon

**Affiliations:** 1 Swansea University Medical School, Swansea University, Swansea, SA2 8PP UK; 2 Department of Animal Sciences, University of Florida, Gainesville, FL 32611 USA

**Keywords:** Cellular microbiology, Mechanisms of disease

## Abstract

Many species of pathogenic bacteria damage tissue cells by secreting toxins that form pores in plasma membranes. Here we show that glucocorticoids increase the intrinsic protection of tissue cells against pore-forming toxins. Dexamethasone protected several cell types against the cholesterol-dependent cytolysin, pyolysin, from *Trueperella pyogenes*. Dexamethasone treatment reduced pyolysin-induced leakage of potassium and lactate dehydrogenase, limited actin cytoskeleton alterations, reduced plasma membrane blebbing, and prevented cytolysis. Hydrocortisone and fluticasone also protected against pyolysin-induced cell damage. Furthermore, dexamethasone protected HeLa and A549 cells against the pore-forming toxins streptolysin O from *Streptococcus pyogenes*, and alpha-hemolysin from *Staphylococcus aureus*. Dexamethasone cytoprotection was not associated with changes in cellular cholesterol or activating mitogen-activated protein kinase (MAPK) cell stress responses. However, cytoprotection was dependent on the glucocorticoid receptor and 3-hydroxy-3-methyl-glutaryl-coenzyme A reductase (HMGCR). Collectively, our findings imply that glucocorticoids could be exploited to limit tissue damage caused by pathogens secreting pore-forming toxins.

## Abstract (web summary)

Treating cell lines with glucocorticoids offers protection against the damage caused by subsequent exposure to selected bacterial pore-forming toxins, and is dependent on the glucocorticoid receptor.

## Introduction

Many species of pathogenic bacteria secrete toxins that form pores in the plasma membrane of eukaryotic cells^1,2^. These pore-forming toxins cause cell damage and cytolysis, which contribute to bacterial virulence and the development of disease. However, treatment with glucocorticoids reduces the severity of some bacterial diseases^3–5^. We assume that these beneficial effects are partly because glucocorticoids restrain inflammation and immunopathology^6^. Here, we explored whether glucocorticoids can also help tissue cells protect themselves against damage caused by pore-forming toxins.

Pore-forming toxins enable the delivery of bacterial virulence factors into eukaryotic cells, result in the leakage of cytosolic molecules that bacteria use as nutrients, and facilitate bacterial invasion^1,2^. Pathogens that secrete pore-forming toxins include *Trueperella pyogenes*, which causes purulent infections in farm animals, *Streptococcus pyogenes*, which causes pharyngitis and impetigo in children, and *Staphylococcus aureus*, which causes pneumonia, sepsis, and skin infections in animals and humans^2,7–10^. *Trueperella pyogenes* and *Streptococcus pyogenes* produce pyolysin and streptolysin O, respectively, which are members of the most common family of pore-forming toxins, the cholesterol-dependent cytolysins. Cholesterol-dependent cytolysins bind plasma membrane cholesterol and then form membrane pores^11,12^. Pyolysin molecules forming 18 nm internal diameter β-barrel transmembrane pores, and streptolysin molecules forming 26 nm diameter pores^9,13,14^. These pores result in potassium efflux from cells, calcium influx, leakage of cytosolic proteins such as lactate dehydrogenase (LDH), actin cytoskeleton alterations, and cytolysis^15–17^. *Staphylococcus aureus* secretes an α-hemolysin that binds to plasma membranes, where 7 molecules oligomerize to form a 1.4 nm internal diameter β-barrel transmembrane pore, which also leads to the leakage of cytosolic molecules and cytolysis^7,18,19^. Preventing or reducing the severity of disease depends on the immune system killing and removing pathogenic bacteria, and on limiting the tissue damage caused by the pathogens and the immune response^20^. Although cells can repair the damage caused by pore-forming toxins^15,21–23^, little is known about the intrinsic protection of cells against these toxins.

Glucocorticoids are secreted to foster metabolic adaptation, inhibit inflammation, and support tissue repair, as part of the stress response to infection^6,24,25^. Glucocorticoids bind to the widely expressed glucocorticoid receptor (GR; encoded by *NR3C1*), which leads to transcription factor repression, gene regulation, and nongenomic effects^24–27^. The pleiotropic effects of glucocorticoids are often cell type and context specific, and include restraining innate immunity, inhibiting mitogen-activated protein kinase (MAPK) stress responses, suppressing the production of inflammatory mediators, and altering cell metabolism^24–27^. Glucocorticoids such as dexamethasone, hydrocortisone and fluticasone are used as anti-inflammatory drugs to limit the inflammation and immunopathology associated with bacterial infections^6,24,25,28^. However, we suggest that glucocorticoids can also stimulate the intrinsic protection of tissue cells against the damage caused by pathogens. If glucocorticoids can increase tissue cell protection against pore-forming toxins this would have important implications for the benefit of using steroid treatments to prevent or limit the pathology caused by infection with pathogenic bacteria.

Here we tested the hypothesis that glucocorticoids help tissue cells protect themselves against the damage caused by pore-forming toxins. We found that treatment with dexamethasone, hydrocortisone or fluticasone protected several types of cells against the damage caused by cholesterol-dependent cytolysins or α-hemolysin. Glucocorticoids increased the intrinsic protection of the cells and cytoprotection was dependent on the glucocorticoid receptor and the rate limiting enzyme in cholesterol biosynthesis, 3-hydroxy-3-methyl-glutaryl-coenzyme A reductase (HMGCR). This previously unrecognized glucocorticoid cytoprotection adds to the established role of glucocorticoids limiting the severity of bacterial diseases by suppressing inflammation and immunopathology.

## Results

### Pyolysin damages HeLa cells

**Fig. 1:**
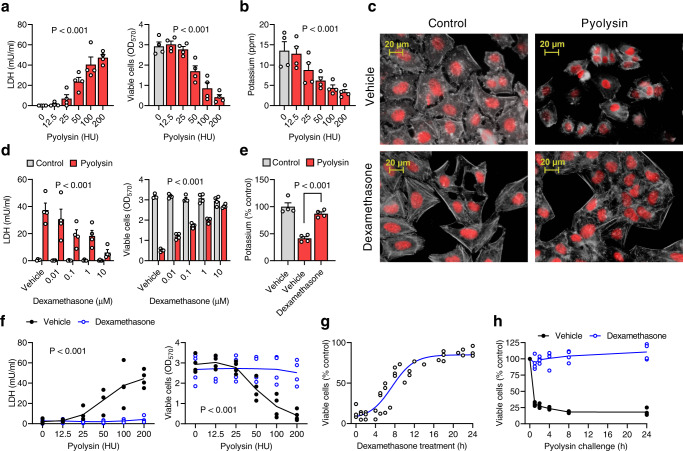
Dexamethasone protects HeLa cells against pyolysin. **a** HeLa cells were challenged with pyolysin and the leakage of LDH into supernatants measured and cell viability determined by MTT assay after 2 h, or **b** intracellular potassium measured after 5 min. Data are mean + s.e.m. from 4 independent experiments; statistical significance was determined using one-way ANOVA. **c** Fluorescent microscope images of cells treated with vehicle or 10 µM dexamethasone for 24 h, then challenged with control or 100 HU/well pyolysin for 2 h, and stained with fluorescent phalloidin (white) and DAPI (red), with images representative of 4 independent experiments. **d** Cells were treated with vehicle or dexamethasone for 24 h, challenged with control or 100 HU/well pyolysin for 2 h, and LDH leakage and cell viability quantified. Data are mean + s.e.m. from 4 independent experiments; statistical significance was determined using two-way ANOVA, and *P* values reported for the treatment effect on the pyolysin challenge. **e** Cells were treated with vehicle or 10 µM dexamethasone for 24 h, challenged with control or 100 HU/well pyolysin for 5 min, and intracellular potassium quantified. Data are mean + s.e.m. from 4 independent experiments; statistical significance was determined using one-way ANOVA with Dunnett’s post hoc test. **f** Cells were treated with vehicle or 10 µM dexamethasone for 24 h, challenged with pyolysin for 2 h, and LDH leakage and cell viability quantified. Data are from 4 independent experiments; statistical significance was determined using two-way ANOVA; lines represent means. **g** Cells were treated with 10 µM dexamethasone for the indicated times, challenged with control or 100 HU/well pyolysin for 2 h, and cell viability quantified. Pyolysin data are percentage of control challenge, with dots representing individual values across 3 independent experiments; the line is the least squares fit. **h** Cells were treated with vehicle or 10 µM dexamethasone for 24 h, challenged with control or 100 HU/well pyolysin for the indicated times, and cell viability determined. Pyolysin data are percentage of control challenge from 4 independent experiments; lines represent means.

Pyolysin is useful for studying cytoprotection because it forms pores in the plasma membrane of many types of eukaryotic cells^8,10^. Furthermore, unlike most cholesterol-dependent cytolysins, pyolysin does not require chemical activation with reducing agents in vitro. We used HeLa cells because these cervical epithelial cells are often used to explore cellular responses to cytolysins^15,16,21,29^. To determine a suitable pyolysin challenge for exploring cytoprotection, we first cultured cells in 24-well plates in serum-free medium for 24 h and then challenged them with a range of amounts of pyolysin for 2 h. Serum-free medium was used to avoid confounding effects of serum cholesterol, corticosteroid-binding globulin, and activation of cell signaling pathways^30^. Cells were challenged with pyolysin for 2 h because this is sufficient time to cause cell damage^10,31^ and because longer challenges might also reflect repair or cell replication. Pyolysin challenge resulted in the leakage of LDH into cell supernatants, and reduced cell viability as determined by MTT assays (Fig. 1a). Pyolysin challenge also resulted in the loss of intracellular potassium (Fig. 1b), altered the actin cytoskeleton and cell shape (upper panels, Fig. 1 c, Supplementary Fig 1a–c), and induced plasma membrane blebbing (Supplementary Fig 1d). We selected 100 HU/well pyolysin for cytoprotection experiments because this caused > 80% cytolysis.

### Dexamethasone protects HeLa cells against pyolysin

We investigated whether treating HeLa cells with dexamethasone for 24 h protected them against a subsequent 2 h pyolysin challenge, without further dexamethasone treatment. Dexamethasone reduced pyolysin-induced LDH leakage and cytolysis (Fig. 1d). As little as 0.01 µM dexamethasone reduced both LDH leakage and cytolysis (*P* < 0.001), and 10 µM dexamethasone reduced LDH leakage by 84%, and reduced cytolysis from 83% to 9%. Treatment with 10 µM dexamethasone also limited pyolysin-induced alterations in cell shape and the actin cytoskeleton (Fig. 1c, Supplementary Fig 1a–c; 28 ± 2% vs 88 ± 2% cells damaged; *n* = 3 independent experiments, Chi-square, *P* < 0.01), and reduced pyolysin-induced blebbing of the plasma membrane (Table. 1). Treatment with 10 µM dexamethasone also reduced loss of intracellular potassium following a 5 min pyolysin challenge (Fig. 1e).

**Table 1:** Quantification of HeLa cell morphology.

Treatment	*n*	Percentage of pyolysin-challenged cells		
		Normal	Blebbed	Fragmented
Vehicle	10	7 ± 2^a^	55 ± 6^a^	38 ± 6^a^
Dexamethasone	7	87 ± 4^b^	8 ± 3^b^	5 ± 2^b^
Hydrocortisone	7	67 ± 13^c^	23 ± 4^c^	10 ± 3^b^

Evaluation of cell shape following treatment of HeLa cells on coverslips with vehicle, 10 µM dexamethasone, or 10 µM hydrocortisone for 24 h, and then challenged with 100 HU/well pyolysin for 2 h. Cells were imaged by phase contrast microscopy, and categorized as normal, blebbed or fragmented, with > 100 cells evaluated across two independent images for each treatment replicate (*n*). Raw data were analyzed by Chi-square with different superscript indicating *P* < 0.05 between treatments, and data presented as mean ± s.e.m. for the pyolysin challenge as a percentage of control challenge.

Dexamethasone was effective against 50 to 200 HU/well pyolysin (Fig. 1f), and cytoprotection was evident when HeLa cells were treated with dexamethasone for ≥ 6 h (Fig. 1g). In addition, treatment with 10 µM dexamethasone for 24 h protected cells against pyolysin challenges lasting between 1 and 24 h (Fig. 1h). Collectively, these data provide evidence for dexamethasone cytoprotection of HeLa cells against pyolysin.

### Dexamethasone protects several types of cells against pyolysin

**Fig. 2:**
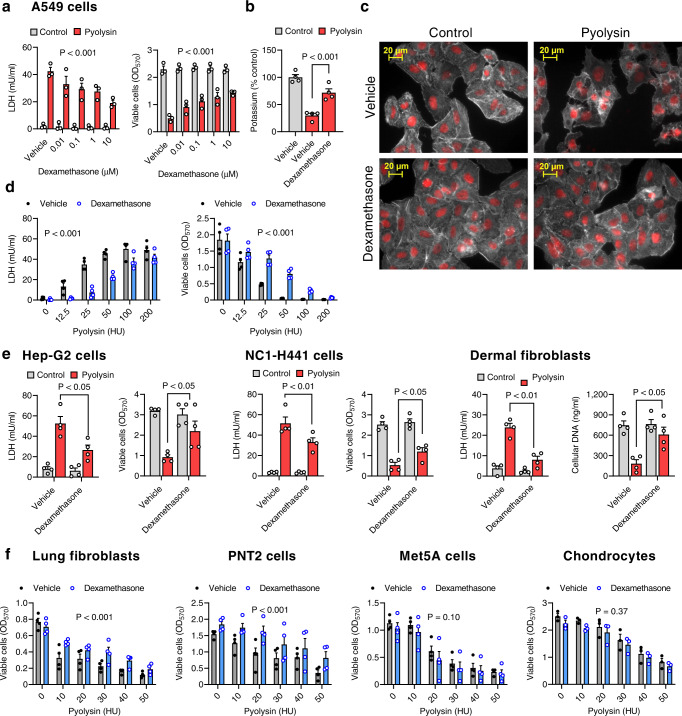
Dexamethasone protects several types of cells against pyolysin. **a** A549 cells were treated with vehicle or dexamethasone for 24 h, challenged with control or 25 HU/well pyolysin for 2 h, and LDH leakage and cell viability quantified. Data are mean + s.e.m. from 3 independent experiments; statistical significance was determined using two-way ANOVA, and P-values reported for the treatment effect on pyolysin challenge. **b** A549 cells were treated with vehicle or 10 µM dexamethasone for 24 h, challenged with control or 25 HU/well pyolysin for 5 min, and intracellular potassium quantified. Data are mean + s.e.m. from 4 independent experiments; statistical significance was determined using one-way ANOVA with Dunnett’s post hoc test. **c** Fluorescent microscope images of A549 cells treated with vehicle or 10 µM dexamethasone for 24 h, challenged with control or 25 HU/well pyolysin for 2 h, and stained with fluorescent phalloidin (white) and DAPI (red); images are representative of 3 independent experiments. **d** A549 cells were treated with vehicle or 10 µM dexamethasone for 24 h, challenged with pyolysin for 2 h, and LDH leakage and cell viability quantified. Data are mean ± s.e.m. from 4 independent experiments; statistical significance was determined using two-way ANOVA. **e** Hep-G2 cells, NCI-H441 cells and dermal fibroblasts were treated with vehicle or 10 µM dexamethasone for 24 h, challenged with control or pyolysin for 2 h (Hep-G2, 100 HU/well; NC1-H441, 200 HU/well; primary human dermal fibroblasts, 25 HU/well), and LDH leakage measured and cell viability quantified by MTT assay or CYQUANT (fibroblasts). Data are mean + s.e.m. from 4 independent experiments; statistical significance was determined using two-way ANOVA, with Bonferroni’s multiple comparison test. **f** Human lung fibroblasts, PNT2 cells, Met5A cells or primary human chondrocytes cells were treated with vehicle or 10 µM dexamethasone for 24 h, challenged with the indicated amounts of pyolysin for 2 h, and cell viability quantified. Data are mean ± s.e.m. from 3 or 4 independent experiments; statistical significance was determined using two-way ANOVA.

To explore if dexamethasone cytoprotection against pyolysin extended beyond HeLa cells, we used A549 lung epithelial cells because they are also frequently used to explore responses to cytolysins^19,32^. Challenging A549 cells with ≥ 25 HU/well pyolysin for 2 h induced LDH leakage and reduced cell viability (ANOVA, *P* < 0.001, Supplementary Fig 2a). However, treating A549 cells with dexamethasone for 24 h reduced LDH leakage and cytolysis following a 2 h challenge with 25 HU/well pyolysin (Fig. 2a), and reduced the loss of intracellular potassium following a 5 min challenge (Fig. 2b). Treatment with 10 µM dexamethasone also reduced pyolysin-induced alterations in cell shape and the actin cytoskeleton (Fig. 2c; 7 ± 4 vs 71 ± 5% cells damaged, *n* = 3 independent experiments, Chi-square, *P* < 0.01). Dexamethasone cytoprotection was effective against 12.5 to 100 HU/well pyolysin (Fig. 2d).

We also tested dexamethasone cytoprotection against pyolysin using a further seven different types of human cells. Leakage of LDH and cytolysis was evident following a 2 h challenge with 100 HU/well pyolysin in Hep-G2 liver cells, 200 HU/well pyolysin in NC1-H441 lung epithelial cells, and 25 HU/well pyolysin in primary normal dermal fibroblasts (Supplementary Fig 2b–d). However, treatment with 10 µM dexamethasone for 24 h reduced LDH leakage and cytolysis following a 2 h pyolysin challenge in Hep-G2 cells, NCI-H441 cells, and dermal fibroblasts (Fig. 2e). Treatment with 10 µM dexamethasone for 24 h also reduced cytolysis following a 2 h challenge with a range of amounts of pyolysin in primary normal human lung fibroblasts, and PNT2 SV40 transformed normal prostate epithelial cells, although not in Met5A SV40 transformed normal mesothelial cells or primary human chondrocytes (Fig. 2f). Finally, treatment with 100 µM dexamethasone for 24 h reduced cytolysis following a 2 h challenge with 200 HU pyolysin in primary bovine endometrial epithelial cells (dexamethasone 48.2 ± 7.8% vs vehicle 82.8 ± 1.2 cytolysis, *P* = 0.015, *n* = 4 independent experiments).

### Glucocorticoids protect cells against pyolysin

**Fig. 3:**
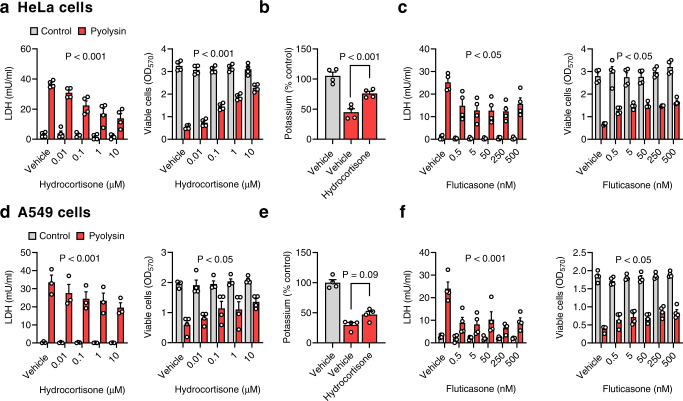
Glucocorticoids protect cells against pyolysin. **a** HeLa cells were treated with vehicle or hydrocortisone for 24 h, challenged with control or 100 HU/well pyolysin for 2 h, and LDH leakage and cell viability quantified. Data are mean + s.e.m. from 4 independent experiments; statistical significance was determined using two-way ANOVA, and *P*-values reported for the treatment effect on the pyolysin challenge. **b** HeLa cells were treated with vehicle or 10 µM hydrocortisone for 24 h, challenged with control or 100 HU/well pyolysin for 5 min, and intracellular potassium quantified. Data are mean + s.e.m. from 4 independent experiments; statistical significance was determined using one-way ANOVA with Dunnett’s post hoc test. **c** HeLa cells were treated with vehicle or fluticasone propionate for 24 h, challenged with control or 100 HU/well pyolysin for 2 h, and LDH leakage and cell viability quantified. Data are mean + s.e.m. from 4 independent experiments; statistical significance was determined using two-way ANOVA, and P-values reported for the treatment effect on the pyolysin challenge. **d**–**f** Experiments a–c, respectively, were repeated using A549 cells challenged with 25 HU/well pyolysin.

We examined whether glucocorticoids other than dexamethasone could protect cells against pyolysin. Treating HeLa cells with hydrocortisone for 24 h reduced LDH leakage and cytolysis following a 2 h pyolysin challenge (Fig. 3a), and reduced the loss of intracellular potassium following a 5 min challenge (Fig. 3b). Furthermore, 10 µM hydrocortisone reduced pyolysin-induced alterations in HeLa cell shape and the actin cytoskeleton (32 ± 9% vs 88 ± 2% cells damaged; *n* = 3 independent experiments, Chi-square, *P* < 0.01), and reduced pyolysin-induced blebbing of the plasma membrane (Table 1). Cytoprotection was evident when cells were treated with hydrocortisone for ≥ 6 h (Supplementary Fig 3a). Treating HeLa cells with the selective glucocorticoid receptor agonist, fluticasone propionate^28^, also reduced LDH leakage and cytolysis following a 2 h pyolysin challenge (Fig. 3c).

Treating A549 cells with hydrocortisone for 24 h reduced LDH leakage and cytolysis following a 2 h pyolysin challenge (Fig. 3d); and tended to reduce the loss of intracellular potassium following a 5 min challenge (Fig. 3e). Furthermore, 10 µM hydrocortisone reduced pyolysin-induced alterations in A549 cell shape and the actin cytoskeleton (16 ± 4 vs 71 ± 5% cells damaged, *n* = 3 independent experiments, Chi-square, *P* < 0.01). Fluticasone also reduced pyolysin-induced LDH leakage and cytolysis in A549 cells (Fig. 3f).

### Dexamethasone cytoprotection against streptolysin O and α-hemolysin

**Fig. 4:**
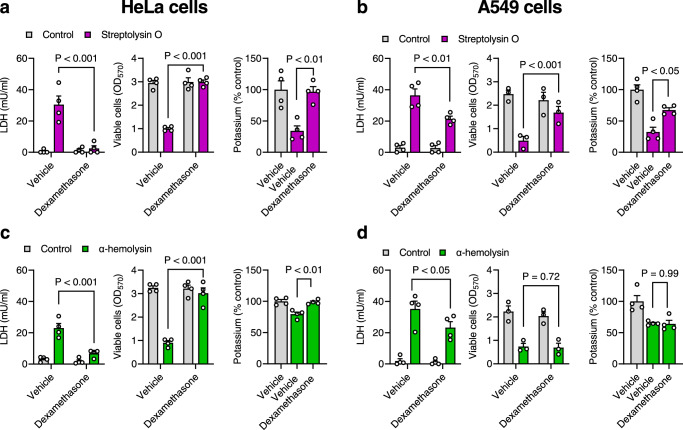
Dexamethasone protects cells against streptolysin O and α-hemolysin. **a**, **b** HeLa cells and A549 cells, respectively, were treated with vehicle or 10 µM dexamethasone for 24 h, challenged with control or streptolysin O (HeLa, 12.5 µg/well; A549, 6.25 µg/well), and LDH leakage and cell viability quantified after 2 h or intracellular potassium measured after 5 min. Data are mean + s.e.m. from 3 or 4 independent experiments; statistical significance was determined using two-way ANOVA, with Šídák’s multiple comparisons test. **c**, **d** HeLa cells and A549 cells, respectively, were treated with vehicle or 10 µM dexamethasone for 24 h, challenged with control or 8 µg/well α-hemolysin, and LDH leakage and cell viability quantified after 24 h or cellular potassium measured after 15 min. Data are mean + s.e.m. from 3 or 4 independent experiments; statistical significance was determined using two-way ANOVA, with Šídák’s multiple comparisons test.

We next explored whether dexamethasone could protect cells against pore-forming toxins other than pyolysin, using streptolysin O and *Staph. aureus* α-hemolysin. Challenging HeLa or A549 cells with streptolysin O resulted in LDH leakage and cytolysis after a 2 h challenge (Supplementary Fig 4a, b). However, treating HeLa cells or A549 cells with 10 µM dexamethasone for 24 h reduced LDH leakage and cytolysis following a 2 h streptolysin O challenge, and reduced the loss of intracellular potassium following a 5 min challenge (Fig. 4a, b). Dexamethasone treatment also reduced LDH leakage and cytolysis following challenge with a range of concentrations of streptolysin O for 2 h (Supplementary Fig 4a, b).

Challenging HeLa or A549 cells with 8 µg/well α-hemolysin resulted in LDH leakage and cytolysis after 24 h (Supplementary Fig 4c, d). However, treating HeLa cells with dexamethasone for 24 h reduced LDH leakage and cytolysis following a subsequent 24 h α-hemolysin challenge, and reduced the loss of intracellular potassium following a 15 min challenge (Fig. 4c, Supplementary Fig 4e). Dexamethasone treatment of A549 cells also reduced α-hemolysin-induced LDH leakage but did not significantly alter cytolysis or loss of intracellular potassium (Fig. 4d, Supplementary Fig 4f). Dexamethasone prevented α-hemolysin-induced alterations in the shape and cytoskeleton of HeLa cells (Supplementary Fig 4g, 17 ± 4 vs 86 ± 3% cells damaged, Chi-square, *n* = 3 independent experiments, *P* < 0.01), but not A549 cells (Supplementary Fig 4h, 89 ± 2 vs 76 ± 6% cells damaged). Taken together, the data in Figs. 1–4 provide evidence for glucocorticoid cytoprotection of eukaryotic tissue cells against pore-forming toxins, and particularly for dexamethasone increasing cell-intrinsic protection against pyolysin.

### Glucocorticoid cytoprotection does not depend on activating MAPK

**Fig. 5:**
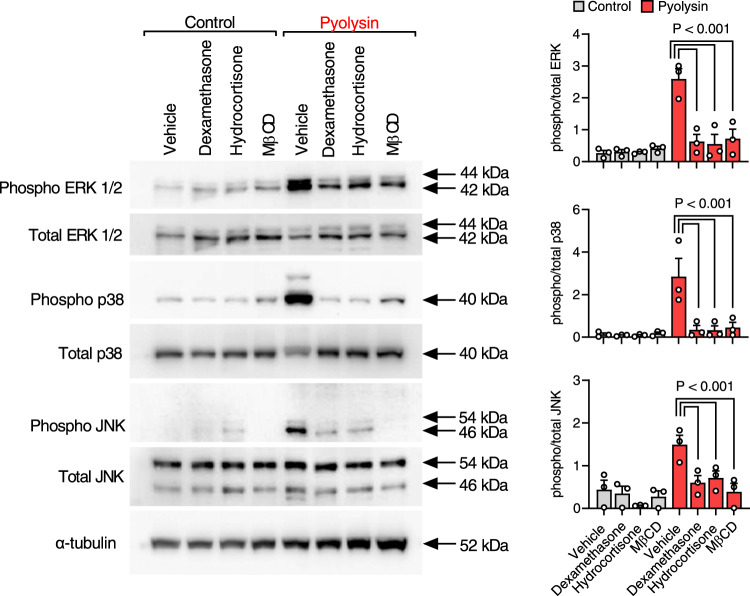
Cytoprotection does not depend on MAPK. Representative Western blot of phosphorylated and total ERK1/2, p38 and JNK, and α-tubulin for HeLa cells treated with vehicle, 10 µM dexamethasone, 10 µM hydrocortisone or 1 mM methyl-β-cyclodextrin (MβCD) for 24 h, and then challenged with control or 100 HU/well pyolysin for 10 min. Densitometry data were normalized to α-tubulin and presented as mean + s.e.m. from 3 independent experiments; statistical significance was determined using ANOVA with Tukey post hoc test.

Pore-forming toxins, including pyolysin, streptolysin O and α-hemolysin, activate the MAPK cell stress response following potassium efflux or calcium influx^15,29,33–35^. Thus, enhancing MAPK activation, cellular potassium efflux or calcium influx are potential mechanisms to protect cells against cytolysins. However, dexamethasone or hydrocortisone did not stimulate ERK1/2, p38 or JNK phosphorylation in HeLa cells (Fig. 5). Instead, glucocorticoid treatment prevented pyolysin-induced phosphorylation of ERK1/2, p38 and JNK, which is consistent with glucocorticoid suppression of MAPK^24,25^, or with preventing pyolysin-induced damage as evidenced by using the cholesterol-depleting agent methyl-β-cyclodextrin^36^. Furthermore, dexamethasone remained cytoprotective when cells were challenged with pyolysin in high-potassium buffer to limit potassium efflux (Supplementary Fig 5a), or in calcium-free conditions to limit calcium influx (Supplementary Fig 5b).

### Glucocorticoid cytoprotection does not depend on reducing cellular cholesterol

**Fig. 6:**
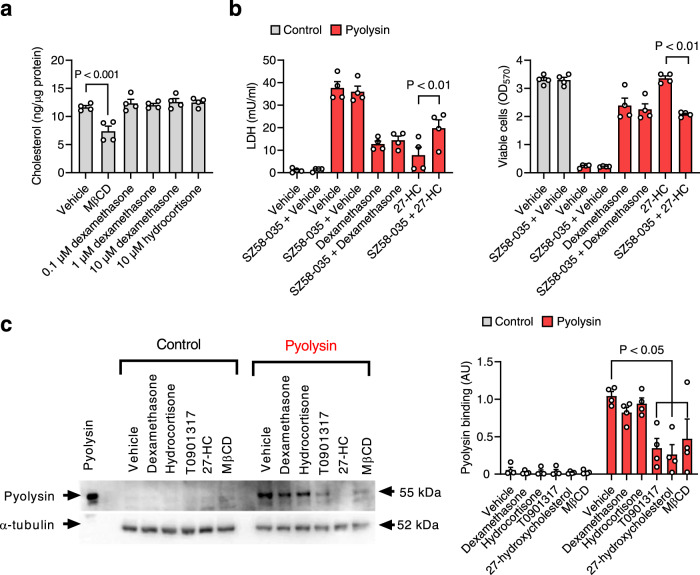
Cytoprotection does not depend on changes in cellular cholesterol. **a** HeLa cells were treated with vehicle, 1 mM methyl-β-cyclodextrin (MβCD), dexamethasone or hydrocortisone for 24 h and total cellular cholesterol measured. Data are mean + s.e.m. from 4 independent experiments; statistical significance was determined using ANOVA with Dunnett’s post hoc test. **b** HeLa cells were cultured with vehicle or 10 µM SZ5-035 for 16 h before treatment with vehicle, 10 µM dexamethasone, or 10 ng/ml 27-hydroxycholesterol (27-HC) for 24 h, in the continuing presence of vehicle or SZ5-035; then challenged with control or 100 HU/well pyolysin for 2 h, and LDH leakage and cell viability quantified. Data are mean + s.e.m. from 4 independent experiments; statistical significance was determined using ANOVA with Bonferroni multiple comparison test. **c** Representative Western blot of pyolysin binding and α-tubulin for HeLa cells treated with vehicle, 10 µM dexamethasone, 10 µM hydrocortisone, 50 nM T0901317, 10 ng/ml 27-hydroxycholesterol (27-HC) or 1 mM methyl-β-cyclodextrin (MβCD) for 24 h, and then challenged with control or 100 HU/well pyolysin for 2 h. Densitometry data (right panel) were normalized to α-tubulin and presented as mean + s.e.m. from 4 experiments; statistical significance was determined using ANOVA with Tukey post hoc test.

Reducing cellular cholesterol protects cells against cholesterol-dependent cytolysins and α-hemolysin^10,17,19^. As expected, treatment with 1 mM methyl-β-cyclodextrin for 24 h reduced total cellular cholesterol in HeLa cells (Fig. 6a), without reducing cell viability (Supplementary Fig 6a). However, treatment with dexamethasone or hydrocortisone did not reduce total HeLa cell cholesterol (Fig. 6a). In A549 cells, the glucocorticoids even increased A549 cell cholesterol (Supplementary Fig 6b).

Cholesterol-dependent cytolysins bind to a labile pool of accessible cholesterol in plasma membranes^11,12^. This is important because side-chain hydroxycholesterols stimulate cholesterol esterification via acyl-coenzyme A:cholesterol acyltransferase (ACAT) to reduce plasma membrane accessible cholesterol, which protects macrophages against cholesterol-dependent cytolysins without reducing total cellular cholesterol, and inhibiting ACAT with SZ58-035 diminishes this protection^37^. Using 27-hydroxycholesterol as a positive control to reduce accessible cholesterol^37^, we also found that culturing HeLa cells with SZ58-035 diminished 27-hydroxycholesterol cytoprotection against a subsequent 2 h cytolysin challenge (Fig. 6b). However, although dexamethasone is also thought to stimulate ACAT^38^, culturing HeLa cells with SZ58-035 did not significantly alter dexamethasone protection against pyolysin-induced LDH leakage or cytolysis (Fig. 6b).

In an independent approach we examined whether glucocorticoids alter plasma membrane-accessible cholesterol by measuring pyolysin binding to HeLa cells^11^. We first verified that treating HeLa cells with a liver-X-receptor agonist (T0901317; Tocris, Abingdon, UK), 27-hydroxycholesterol, or methyl-β-cyclodextrin diminished pyolysin binding (Fig. 6c). However, treating cells with dexamethasone or hydrocortisone had no significant effect on pyolysin binding. Collectively, these results suggest that glucocorticoid cytoprotection was not mediated by reducing plasma membrane-accessible cholesterol.

### Serum impairs glucocorticoid cytoprotection

**Fig. 7:**
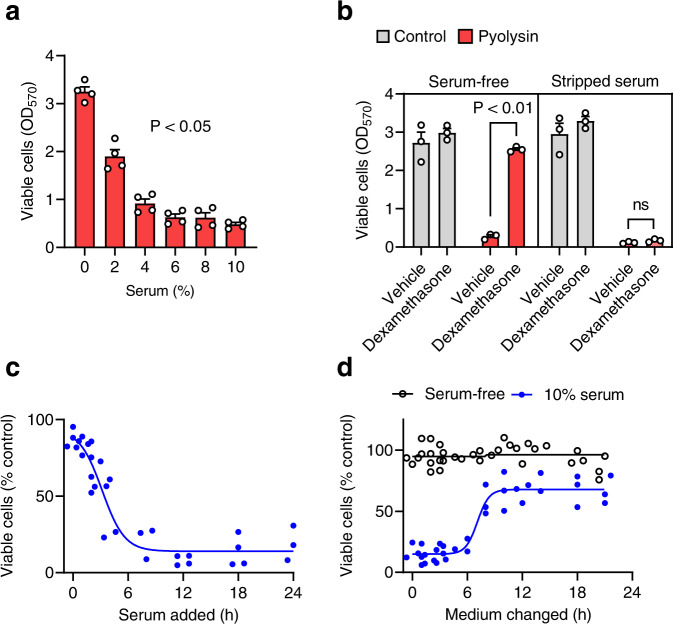
Serum impairs glucocorticoid cytoprotection. **a** HeLa cells were treated with 10 µM dexamethasone for 24 h in medium containing the indicated serum concentrations, then challenged with 100 HU/well pyolysin for 2 h, and cell viability quantified by MTT assay. Data are mean + s.e.m. from 4 independent experiments; statistical significance was determined using ANOVA. **b** HeLa cells were treated with vehicle or 10 µM dexamethasone for 24 h in medium with or without 10% stripped serum, then challenged with control or 100 HU/well pyolysin for 2 h, and cell viability quantified. Data are mean + s.e.m. from 3 independent experiments; statistical significance was determined using ANOVA with Bonferroni multiple comparison test. **c** HeLa cells were treated with vehicle or 10 µM dexamethasone for 24 h in medium supplemented with 10% serum at the indicated time prior to being challenged with control or 100 HU/well pyolysin for 2 h, and cell viability quantified. Data are percentage viability of control challenge, dots represent individual values from 4 independent experiments, and the line is the least squares fit. **d** HeLa cells were treated with 10 µM dexamethasone in medium with or without 10% serum, then at the indicated time points supernatants were discarded and the medium changed to serum-free medium for the remainder of the 24 h, before the cells were challenged with control or 100 HU/well pyolysin for 2 h, and cell viability quantified. Data are percentage viable cells compared with the control challenge, dots represent individual values from 3 independent experiments, and lines are least squares fits.

We used serum-free medium in our experiments to avoid confounding effects of serum cholesterol, steroids, corticosteroid-binding globulin, and LDH, and to avoid serum-activation of gene and cell signaling pathways^30,39^. Indeed, glucocorticoid cytoprotection against pyolysin was prevented by culturing HeLa cells in ≥ 6% fetal bovine serum (Fig. 7a) or by culturing A549 cells with 10% serum (Supplementary Fig 7). Even culturing HeLa cells with 10% charcoal-stripped serum, which is cholesterol and steroid-depleted^40^, prevented dexamethasone cytoprotection against pyolysin (Fig. 7b). Furthermore, when HeLa cells cultured in serum-free media were treated with dexamethasone for 24 h, cytoprotection was abolished if 10% serum was added for ≥ 6 h before the pyolysin challenge (Fig. 7c; ANOVA, Dunnett post hoc test, *P* < 0.05). Conversely, cytoprotection was restored by replacing medium containing 10% serum and 10 µM dexamethasone, with serum-free medium without dexamethasone for ≥ 8 h before a pyolysin challenge (Fig. 7d; ANOVA, Dunnett post hoc test, *P* < 0.05). Collectively, these data provide evidence that serum can reversibly diminish glucocorticoid cytoprotection against pyolysin.

### Cytoprotection depends on the glucocorticoid receptor

**Fig. 8:**
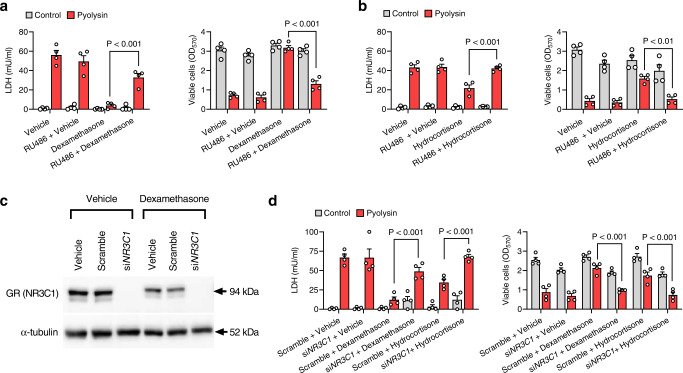
Cytoprotection depends on the glucocorticoid receptor. **a** HeLa cells were treated with vehicle or 10 µM dexamethasone, or **b** 10 µM hydrocortisone, in medium with or without 10 µM RU486 for 24 h, then challenged with control or 100 HU/well pyolysin for 2 h, and LDH leakage and cell viability quantified. Data are mean + s.e.m. of 4 independent experiments; statistical significance was determined using two-way ANOVA and Bonferroni post hoc test. **c** Western blots of glucocorticoid receptor (GR) and α-tubulin for HeLa cells transfected with scrambled siRNA or siRNA targeting *NR3C1*; images are representative of 4 independent experiments. **d** HeLa cells were transfected for 48 h with scramble siRNA or siRNA targeting *NR3C1*, treated with vehicle, 10 µM dexamethasone or 10 µM hydrocortisone for 24 h, then challenged with control or 100 HU/well pyolysin for 2 h, and LDH leakage and cell viability quantified. Data are mean + s.e.m. from 4 independent experiments; statistical significance was determined using two-way ANOVA and Bonferroni post hoc test.

We next investigated whether the glucocorticoid receptor was important for cytoprotection because glucocorticoids can also repress transcription factors and exert nongenomic effects^24–27^. Culturing HeLa or A549 cells with the glucocorticoid receptor antagonist RU486^41^, diminished dexamethasone and hydrocortisone cytoprotection against pyolysin (Fig. 8a, b; Supplementary Fig 8). Similarly, using siRNA targeting *NR3C1* to deplete the glucocorticoid receptor in HeLa cells (Fig. 8c), diminished dexamethasone and hydrocortisone cytoprotection against pyolysin (Fig. 8d). Together these experiments provide evidence that dexamethasone and hydrocortisone cytoprotection against pyolysin was dependent on the glucocorticoid receptor.

**Fig. 9:**
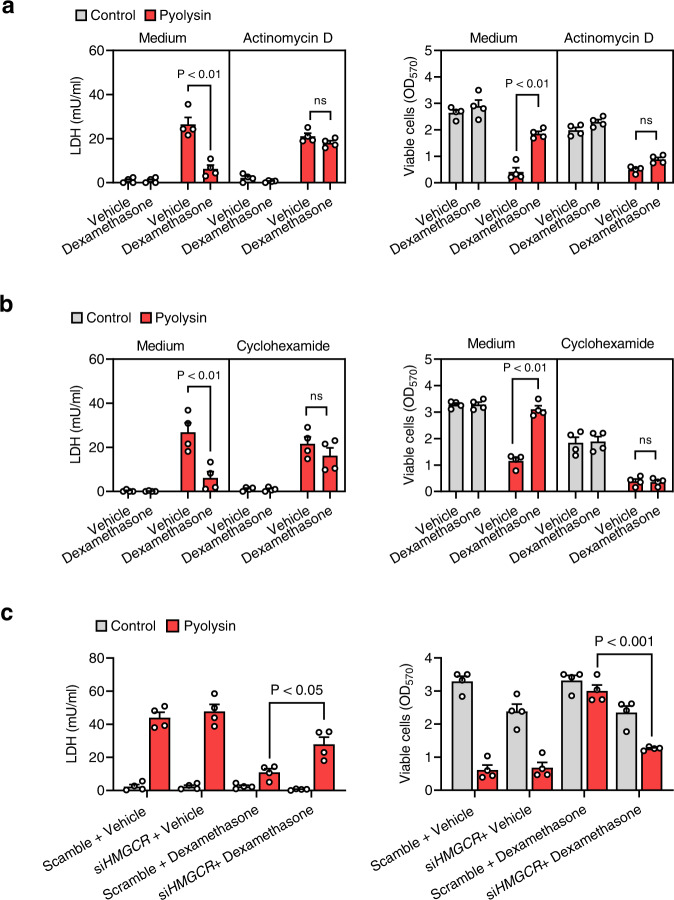
Glucocorticoid cytoprotection is partially reliant on HMGCR. **a** HeLa cells were cultured in medium or with 1 µg/ml cycloheximide or **b** 0.25 µg/ml actinomycin D, and treated with vehicle or 10 µM dexamethasone for 24 h, and then challenged with control medium or 100 HU pyolysin for 2 h. The leakage of LDH into supernatants was measured after 2 h, and viable cells determined by MTT assay. Data are presented as mean + s.e.m. from 4 independent experiments; statistical significance was determined using two-way ANOVA with Bonferroni multiple comparison test. **c** HeLa cells were transfected for 48 h with scramble siRNA or siRNA targeting *HMGCR*, treated with vehicle or 10 µM dexamethasone for 24 h, then challenged with control medium or 100 HU pyolysin for 2 h. The leakage of LDH into supernatants was measured after 2 h, and viable cells determined by MTT assay. Data are presented as mean + s.e.m. from 4 independent experiments; statistical significance was determined using two-way ANOVA and Bonferroni multiple comparison test.

We finally considered whether cytoprotection against pyolysin might depend on the glucocorticoid receptor activating a specific gene because cytoprotection required dexamethasone treatment for ≥ 6 h (Fig. 1g), and cytoprotection was precluded when cells were cultured with actinomycin to inhibit gene transcription (Fig. 9a) or with cycloheximide to inhibit translation (Fig. 9b). As serum reversibly prevented glucocorticoid cytoprotection, we searched the literature for genes regulated in an opposing manner by glucocorticoids and serum. Although glucocorticoids and serum regulate numerous genes^26,30,39^, we identified 19 genes regulated by both dexamethasone and serum, of which only 11 were regulated in an opposing manner (Supplemental Table 1). Whilst most of these genes seemed mechanistically unlikely to contribute to cytoprotection, a potential candidate was *HMGCR*, which encodes the rate-limiting enzyme in the mevalonate pathway for cholesterol biosynthesis^42^. Glucocorticoids increase the expression of *HMGCR*, whilst serum reduces the expression of *HMGCR*^43^. Using siRNA to target *HMGCR* in HeLa cells diminished dexamethasone cytoprotection against pyolysin (Fig. 9c, Supplementary Fig 9). Thus, we conclude that dexamethasone cytoprotection is at least partially reliant on HMGCR.

## Discussion

We found evidence for a previously unidentified cytoprotective effect of glucocorticoids against pore-forming toxins. Pyolysin, streptolysin O and α-hemolysin formed pores in HeLa cell plasma membranes, as determined by leakage of potassium and LDH from cells, activation of the MAPK stress response, changes in cell shape and the cytoskeleton, and induction of cytolysis. These observations are typical of the effects of pore-forming toxins on cells^1,2,7,15,34^. However, we found that treating HeLa cells with dexamethasone or hydrocortisone reduced cytolysin-induced potassium and LDH leakage, prevented pyolysin-induced phosphorylation of ERK, p38 and JNK, limited cytoskeletal changes, and prevented cytolysis. Using our LDH leakage data, we estimate the EC_50_ to be 0.09 µM dexamethasone for HeLa cell protection against pyolysin. The level of cytoprotection was striking, with 10 µM dexamethasone providing > 90% protection.

Glucocorticoid cytoprotection against pyolysin extended to several types of tissue cells, but the level of cytoprotection varied between the cell types. This variation in cytoprotection might reflect differences in glucocorticoid responses, which are often cell-type and context specific^24–27^. Alternatively, the variation in cytoprotection might reflect differences in the cell sensitivity to pyolysin, which has been noted previously amongst different cell lines, and between epithelial and stromal cells^10,36^. We also found differences in cytoprotection between the cholesterol-dependent cytolysins and α-hemolysin, which may reflect the more than ten-fold smaller diameter of the α-hemolysin pores^14,18^.

Protection implies defending or guarding against attack, injury, or damage. Whereas repair encompasses replacing or fixing damaged, worn, or faulty structures. Recognized repair mechanisms for cells damaged by pore-forming toxins include endocytosis, exocytosis, MAPK, the unfolded protein response, and caspase activity^2,15,22,35^. These mechanisms are usually initiated by potassium efflux or calcium influx, but glucocorticoid cytoprotection did not depend on potassium or calcium concentrations in the present study. In addition, glucocorticoids suppress MAPK phosphorylation;^25,39^ whereas, activating MAPK is associated with reducing the damage caused by pore-forming toxins^15,34,35^. Consistent with glucocorticoids stimulating actin stress fiber formation and stabilizing the cytoskeleton^44^, we also noted that glucocorticoids stimulated the formation of actin stress fibers in HeLa and A549 cells. These changes might limit cytolysin-induced cytoskeletal changes; although, it is unclear how this would limit the leakage of potassium or LDH. Indeed, a notable feature was that pyolysin bound to cells, but dexamethasone treatment appeared to limit the formation of pores, as determined by leakage of potassium or LDH. The ability of glucocorticoid treatment to limit cytolysin-induced potassium leakage implies that the early stages of pore formation were prevented, as well as the later stages of membrane damage and cytolysis, which was evidenced by LDH leakage.

Dexamethasone cytoprotection against pyolysin was unexpected because glucocorticoids are not recognized cholesterol-lowering agents, unlike statins, interferons, oxysterols or cyclodextrins, which have been used to limit the cell damage or binding of cholesterol-dependent cytolysins^10–12,19,29,37,45^. There is a pool of labile cholesterol accessible to cholesterol-dependent cytolysins when cholesterol comprises > 35 mol% of membrane lipids^11,12,37^. Changes in cholesterol synthesis, uptake, efflux, or metabolism alter the ability of cholesterol-dependent cytolysins to bind to cells. However, in the present study, we found that glucocorticoids did not reduce the total cellular cholesterol or the accessible pool of plasma membrane cholesterol. These findings agree with previous observations that dexamethasone stimulates cholesterol synthesis and suppresses cellular cholesterol efflux^46^. Although dexamethasone can stimulate ACAT^38^, we also found that, unlike side-chain hydroxycholesterols^37^, glucocorticoids did not significantly protect cells by increasing cholesterol esterification. We therefore reason that reduced plasma membrane cholesterol was unlikely to be a mechanism for glucocorticoid cytoprotection. However, other changes in the plasma membrane might prevent pore-formation without altering total cellular cholesterol. Interestingly, cortisol increases hepatocyte plasma membrane fluidity and alters the membrane order in rainbow trout^47^. The packing of the phospholipids in plasma membranes alters the distribution and accessibility of cholesterol, and less tight packing of phospholipids can be exploited to promote cholesterol-dependent binding^48,49^. Such changes in lipid order also have wider effects on the sensitivity of membranes to damage. For example, a high lipid order in killer T cells helps to protect the plasma membrane against damage by repelling the host pore-forming toxin, perforin^50^.

Although glucocorticoids can repress transcription factors and have rapid nongenomic effects, they usually act by binding to the glucocorticoid receptor in the cytosol^24–27^. The receptor then moves to the nucleus and binds glucocorticoid response elements leading to transactivation or transrepression of genes. We thought that the cytoprotection mechanism was likely to be a glucocorticoid receptor-dependent effect because cytoprotection required glucocorticoid treatment for ≥ 6 h, and inhibiting transcription or translation diminished cytoprotection. The glucocorticoid receptor inhibitor RU486 also diminished dexamethasone cytoprotection. One concern might be that RU486 also has anti-progestin activity, but HeLa cells do not express the progesterone receptor^51^. In addition, using siRNA targeting *NR3C1* to reduced glucocorticoid receptor protein diminished dexamethasone cytoprotection. Collectively, these findings provide evidence that glucocorticoid cytoprotection against pore-forming toxins was dependent on the glucocorticoid receptor.

Glucocorticoids are used as an adjunct therapy for some bacterial infections because they reduce inflammation and immunopathology^6,24,25^. Our findings imply that glucocorticoids might also prophylactically protect against tissue damage caused by bacteria that secrete pore-forming toxins. A future challenge will be to disentangle the anti-inflammatory and protective roles of glucocorticoids. Another challenge is that glucocorticoids might have also altered starvation responses in the cells to affect their susceptibility to pore-forming toxins. We did not find evidence for glucocorticoid treatment altering the number of viable cells with or without serum using MTT assays, cellular DNA assays, or cytology. However, our unexpected finding that culturing cells with serum reversibly diminished glucocorticoid cytoprotection inspired a search for factors that are regulated in an opposing manner by serum and glucocorticoids. Although serum and glucocorticoids regulate a wide range of genes and pathways^25,26,30^, few are differentially regulated. One example was that *HMGCR* expression is repressed by serum treatment^30^, whereas glucocorticoids increase HMGCR activity in HeLa cells^43^. Strikingly, we found that using siRNA to deplete HMGCR diminished glucocorticoid cytoprotection against pyolysin. How HMGCR mechanistically links glucocorticoids with cytoprotection warrants further investigation.

Whether glucocorticoid cytoprotection is evident in vivo also warrants further investigation. A potential limitation for translating our findings is that whilst short-term glucocorticoid treatment is well tolerated in patients, prolonged use or high doses of glucocorticoids are associated with adverse endocrine or immunosuppression side-effects^25^. This has led to the development of selective glucocorticoid receptor agonists, such as fluticasone, with a high affinity for the glucocorticoid receptor and improved anti-inflammatory actions^28^. Our findings may now stimulate a search for selective glucocorticoid receptor agonists that also improve cytoprotection, which would help patients better tolerate pathogens^20^.

In summary, we found that treatment with glucocorticoids protected several types of cells against pore-forming toxins. Glucocorticoids increased HeLa and A549 cell-intrinsic protection against damage and cytolysis caused by pyolysin, streptolysin O and α-hemolysin. This glucocorticoid cytoprotection was dependent on the glucocorticoid receptor. Our findings imply that glucocorticoids could be exploited to help tissues better tolerate the presence of pathogenic bacteria that secrete pore-forming toxins.

## Materials and methods

### Cell culture

Human cervical epithelial HeLa cells (CCL2, 93021013; European Collection of Authenticated Cell Cultures (ECACC), Public Health England, Salisbury, UK), were cultured in complete medium comprising DMEM (Thermo Fisher Scientific, Paisley, UK), 10% heat-inactivated fetal bovine serum (FBS, Biosera, East Sussex, UK), 2 mM L-glutamine (Thermo Fisher Scientific), and 1% Antibiotic, Antimycotic Solution (ABAM; Merck, Gillingham, UK). Human lung epithelial A549 cells (CCL-185; ATCC, Middlesex, UK) were cultured in complete medium comprising RPMI 1640, 10% FBS, 2 mM L-glutamine (Thermo Fisher Scientific), and 1% ABAM. Human liver epithelial HepG2 cells (HB-8065; ATCC) were cultured in complete medium comprising DMEM, 10% FBS, 2 mM L-Glutamine, and 1% ABAM. Human lung epithelial NCI-H441 cells (HTB-174; ATCC) were cultured in complete medium comprising RPMI 1640, 10% FBS, 2 mM L-glutamine, and 1% ABAM.

Primary normal human dermal fibroblasts (C-12302; Promocell, Heidelberg, Germany) were obtained from the dermis of normal adult skin. Cells were cultured in Fibroblast Growth Medium 2 with Supplement mix (Promocell). Primary normal human lung fibroblasts were obtained from normal healthy human lung parenchyma (HLF, PCS-201-013; ATCC). Cells were cultured in complete medium comprising low glucose DMEM (Thermo Fisher Scientific), 10% FBS, 2 mM L-glutamine, and 1% ABAM.

Normal human prostate PNT2 cells (95012613; ECACC) were isolated from a normal individual and immortalized with SV40. Normal human mesothelial MET5A cells (CRL-9444; ATCC) were isolated from pleural fluids obtained from non-cancerous individuals and immortalized with the pRSV-T plasmid. Both cells were cultured in complete medium comprising RPMI 1640, 10% FBS, 2 mM L-glutamine, and 1% ABAM.

Adult primary human chondrocytes were isolated from waste nasoseptal surgical tissue collected with informed consent from healthy donors undergoing septorhinoplasty at Singleton and Morriston Hospitals, Swansea, UK, with approval from the Swansea Bay University Health Board (IRAS ID 99202). Primary human chondrocytes were isolated by sequential digestion of nasoseptal cartilage tissue with 0.2% (w/v) pronase (Merck) for 40 min followed by 0.24% (w/v) collagenase solution (Merck) for 16 h^52^. Cells were cultured in complete medium comprising DMEM, 10% FBS, 2 mM L-glutamine, 1 mM D-glucose, 0.1% MEM NEAA (Merck), and 1% ABAM.

Primary bovine endometrial epithelial cells were isolated from normal female genital tracts collected from cattle after they were slaughtered during the normal work of a commercial slaughterhouse, with approval from the United Kingdom Department for Environment, Food and Rural Affairs under the animal by-products regulation (EC) No. 1069/2009 (registration number U1268379/ABP/OTHER). Briefly, epithelial cells were isolated by dissecting the endometrium and incubating chopped tissue for 1 h at 37°C with gentle agitation in a digestive solution (375 BAEE units trypsin ethylenediaminetetra-acetic acid, 50 mg collagenase II, 100 mg bovine serum albumin (BSA) and 10 mg DNase I in 100 ml Hanks balanced salt solution; all Merck)^10^. The resultant cell suspension was sieved through a 40-μm mesh (Cambridge Bioscience, Cambridge, UK) and the epithelial cells were then collected by back-flushing the mesh. The endometrial epithelial cells were cultured in complete medium comprising RPMI 1640, 10% FBS, 2 mM L-glutamine, and 1% ABAM.

Cells were cultured in 75 cm^2^ flasks (Greiner Bio-One) at 37.5°C in humidified air with 5% carbon dioxide, and the medium replenished every 48–72 h. For experiments, cells were seeded in 24-well tissue culture plates (TPP, Trasadingen, Switzerland), using 1 ml/well complete medium, and cultured until 70% confluent.

### Pore-forming toxins

Pyolysin protein (628,338 HU/mg protein) was generated and purified from the *plo* plasmid pGS59^10,13^. Streptolysin O was stored as 1 mg/ml solution and activated with 10 mM dithiothreitol, and *Staphylococcus aureus* α-hemolysin was stored as 0.5 mg/ml solution according to the manufacturer’s instructions (all Merck).

### Experiments

Pyolysin challenge concentrations were determined by culturing cells for 24 h in serum-free medium and then challenging with 12.5–400 HU pyolysin in 200 µl/well Dulbecco’s PBS (DPBS, Thermo Fisher Scientific) for 15 min, which was then supplemented with 400 µl/well serum-free medium for 1 h 45 min. At the end of the challenge period, supernatants were collected to measure LDH leakage, and cell viability was measured using an MTT assay.

To examine whether glucocorticoids protected cells against pyolysin, cells were incubated in serum-free media containing 0.1% DMSO vehicle or the range of concentrations specified in *Results* of dexamethasone (#D4902; Merck), hydrocortisone (#H0888; Merck), or fluticasone propionate (#2007; Tocris, Abingdon, UK), which were based on the manufacturers’ instructions and publications^28,53,54^. The 24 h treatment period was based on work investigating cytoprotection;^10,19,31^ except for experiments exploring the duration of glucocorticoid treatment required to protect cells against pore-forming toxins, where HeLa cells were treated with 10 µM dexamethasone or hydrocortisone for a range of times up to 24 h. After the treatment period, supernatants were discarded, and cells were challenged with control or pyolysin as specified in *Results*. To determine whether cytoprotection extended beyond pyolysin, HeLa and A549 cells were treated with dexamethasone for 24 h, and then challenged with control or streptolysin O for 2 h, or α-hemolysin for 24 h as specified in *Results*. The α-hemolysin challenge concentration range was based on previous work^32^, and the 24 h duration was selected following preliminary experiments. At the end of the challenge periods, supernatants were collected to measure LDH leakage, and cell viability was evaluated by MTT assay or transmitted light micrographs were captured using an Axiocam ERc 5s microscope camera (Zeiss), and the proportion of normal, blebbing, and fragmented cells were counted using two independent images per treatment replicate.

To explore the effects of glucocorticoid treatment on pyolysin-induced MAPK signaling, HeLa cells were seeded at 1.5 × 10^5^ cells per well on 6-well plates in complete medium for 24 h, then treated with serum-free medium or medium containing 10 µM dexamethasone, 10 µM hydrocortisone, or 1 mM methyl-β-cyclodextrin (Merck) for 24 h, and then challenged with control serum-free medium or medium containing 100 HU/well pyolysin for 10 min. and MAPK evaluated by Western blotting (see below).

To examine the effect of potassium on cytoprotection, HeLa cells were cultured for 24 h in medium containing vehicle or 10 µM dexamethasone, the cells were washed twice with buffer, and then challenged with 100 HU/well pyolysin in 200 µl/well low-potassium buffer (140 mM NaCl, 5 mM KCl, 10 mM HEPES, 1.3 mM CaCl_2_, 0.5 mM MgCl_2_, 0.36 mM K_2_HPO_4_, 0.44 mM KH_2_PO_4_, 5.5 mM D-glucose, 4.2 mM NaHCO_3_) or high-potassium buffer (5 mM NaCl, 140 mM KCl, 10 mM HEPES, 1.3 mM CaCl_2_, 0.5 mM MgCl_2_, 0.36 mM K_2_HPO_4_, 0.44 mM KH_2_PO_4_, 5.5 mM D-glucose, 4.2 mM NaHCO_3_) for 2 h^21^. To examine the effect of calcium on cytoprotection, HeLa cells were cultured for 24 h in medium containing vehicle or 10 µM dexamethasone, the cells were washed twice with calcium-free DPBS, and then challenged with 100 HU/well pyolysin in 200 µl/well calcium-free or calcium-containing DPBS for 15 min, supplemented with 400 µl of serum-free medium with or without calcium for 1 h 45 min. Supernatants were collected to measure LDH leakage, and cell viability was measured using an MTT assay.

To examine the role of ACAT in glucocorticoid cytoprotection against pyolysin we used the selective ACAT inhibitor Sandoz 58-035 (SZ58-035, Merck)^37,55^. HeLa cells were washed twice with phosphate-buffered saline (PBS, Thermo Fisher Scientific), then cultured with 0.1% DMSO vehicle or 10 µM SZ5-035 in serum-free culture medium. After a 16 h incubation, cells were washed twice with PBS, then incubated for 24 h in serum-free culture medium containing 10 ng/ml 27-hydroxycholesterol (Avanti Polar Lipids, Alabama, United States) or 10 µM dexamethasone, in combination with vehicle or 10 µM SZ5-035. Cells were challenged with control or pyolysin for 2 h, and then supernatants were collected to measure LDH leakage, and cell viability was measured using an MTT assay.

To investigate the effect of glucocorticoid treatment on the abundance of membrane-bound pyolysin, HeLa cells were seeded at 1.5×10^5^ cells/well on 6-well plates in complete medium for 24 h, and then treated in serum-free medium or medium containing 10 µM dexamethasone, 10 µM hydrocortisone, 50 nM T0901317, 10 ng/ml 27 hydroxycholesterol or 1 mM methyl-β-cyclodextrin for 24 h, and then challenged with control serum-free medium or medium containing 100 HU/well pyolysin for 2 h, and pyolysin binding determined by Western blotting.

To explore the effect of serum on cytoprotection, HeLa cells were treated with dexamethasone in medium containing heat-inactivated FBS or charcoal-stripped heat-inactivated FBS for the durations specified in Results. To investigate whether serum effects on cytoprotection were reversible, cells were treated with 10 µM dexamethasone in medium with or without 10% FBS, and then at a range of time points from 1 to 24 h, supernatants were discarded, cells were washed twice with PBS, and the medium changed to serum-free medium for the remainder of the 24 h period, before the cells were challenged with control or pyolysin for 2 h, and cell viability was measured using an MTT assay.

To examine the role of the glucocorticoid receptor in cytoprotection, we used the inhibitors actinomycin D, cycloheximide, and RU486 (all Merck). Cells were incubated in serum-free medium with vehicle, 10 µM dexamethasone, or 10 µM hydrocortisone, in combination with 0.1% DMSO solvent, 10 µM RU486, or 1 µg/ml cycloheximide as specified in *Results*. Alternatively, cells were treated with 0.15% DMSO solvent or 0.25 µg/ml actinomycin D for 2 h, then treated with the combinations of vehicle, 10 µM dexamethasone, and actinomycin D for 24 h as specified in Results. Supernatants were discarded, the cells were challenged with control or pyolysin for 2 h, and supernatants were collected to measure LDH leakage, and cell viability was measured using an MTT assay.

To examine the role of the glucocorticoid receptor and HMGCR in cytoprotection, siRNA was used to knockdown the expression of *NR3C1* or *HMGCR*, respectively. Cells were transfected with scramble ON-TARGET Plus non-targeting siRNAs (#D-001810-0X; Horizon discovery, Cambridge, UK), ON-TARGET Plus SMART pool siRNAs targeting *NR3C1* (L-003424-00-0005; Horizon discovery), or siRNA designed using Dharmacon siDesign Centre to target *HMGCR* (sense, GGAUAAACCGAGAAAGAAAUU; antisense, UUUCUUUCUCGGUUUAUCC). In 24-well culture plates, 50 pmol of siRNA was added to 100 µl/well Opti-MEM 1 medium with 1.5 µl Lipofectamine RNAiMAX Reagent (Thermo Fisher Scientific) and incubated for 20 min at room temperature. HeLa cells were then seeded with 4 ×10^4^ cells in 900 µl DMEM medium with 10% FBS for 48 h. Cells were then treated with vehicle, 10 µM dexamethasone or 10 µM hydrocortisone for 24 h in serum-free culture medium prior to a 2 h challenge with control medium or medium containing 100 HU pyolysin. At the end of the challenge period, supernatants were collected to measure LDH leakage and cell viability was evaluated using MTT assays.

### Cell viability

The viability of cells was assessed by the mitochondria-dependent reduction of 3-(4,5-dimethylthiazol-2-yl)-2,5-diphenyltetrazolium bromide (MTT, Merck)^10,56^. Cells were incubated in 250 µl/well serum-free culture media containing 1 mg/ml MTT for 2 h. The medium was then discarded, and cells were lysed with 300 µl/well dimethyl sulfoxide (Merck). Optical density (OD_570_) was measured using a POLARstar Omega microplate reader (POLARstar Omega; BMG, Labtech, Offenburg, Germany). The viability of dermal fibroblasts was assessed by quantifying cellular DNA using the CyQUANT Cell Proliferation Assay Kit (Thermo Fisher Scientific) according to the manufacturer’s instructions.

### Lactate dehydrogenase

Lactate dehydrogenase activity was quantified by measuring the LDH-dependent conversion of lactate to pyruvate, via the reduction of nicotinamide adenine dinucleotide (NAD^+^) to NADH, which is detected by the NADH-dependent reduction of a tetrazolium salt to formazan^17^. Briefly, 0–20 nmol/well NADH standard (Merck) or 20 µl/well cell supernatant was added to 30 µl/well assay buffer (0.2 M Tris hydrochloride, pH 8.2, Merck) in duplicate in clear half-area 96 well plates (Greiner). A reaction mixture (54 mM sodium L-lactate, 0.66 mM 2-p-iodophenyl-3-p-nitrophenyl tetrazolium chloride, 0.28 mM phenazine methosulfate and 1.3 mM NAD^+^; all Merck) was prepared in assay buffer, and 50 µl added to each well. The optical density at 570 nm was measured immediately (A_0_) and after 30 min incubation at 37.5°C (A_30_), using a POLARstar Omega plate reader. The difference in supernatant optical density (A_30_ - A_0_) was used to calculate the amount of NADH generated from the NADH standards. The supernatant LDH activity in mU/ml was then calculated as: LDH activity = nmole NADH / (30 min reaction time × 0.02 ml volume supernatant). The inter and intra-assay coefficients of variation were < 8% and < 5%, respectively.

### Potassium

To quantify the leakage of potassium cells were seeded in complete medium at 1.5 × 10^5^ cells per well in 6-well culture plates and incubated for 24 h, before treatment with vehicle, 10 µM dexamethasone or 10 µM hydrocortisone in serum-free medium for 24 h. Supernatants were discarded, and the cells were washed 3 times with potassium-free choline buffer (129 mM choline-Cl, 0.8 mM MgCl_2_, 1.5 mM CaCl_2_, 5 mM citric acid, 5.6 mM glucose, 10 mM NH_4_Cl, 5 mM H_3_PO_4_, pH 7.4; all Merck), and then challenged with choline buffer, pyolysin or streptolysin O for 5 min, or with 8 µg/5 × 10^4^ cells α-hemolysin for 15 min (no potassium leakage was detectable from HeLa cells after 5 min of α-hemolysin challenge). Cells were then washed 3 times in ice-cold choline buffer and lysed in 0.5% Triton X-100 (Merck) in distilled water for 20 min on a rocker at room temperature. Potassium was measured in supernatants and lysates using a Jenway PFP7 flame photometer (Cole-Parmer, Stone, Staffordshire, UK). The inter- and intra-assay coefficients of variation were < 4%.

### Cholesterol

To quantify cellular cholesterol in HeLa and A549 cells, 1.5 × 10^5^ cells per well were seeded on 6-well culture plates in complete medium for 24 h. Cells were treated in serum-free medium for a further 24 h with vehicle, 10 µM dexamethasone, 10 µM hydrocortisone or 1 mM methyl-β-cyclodextrin, before the cells were washed twice with PBS, collected in 200 µl/ well cholesterol assay buffer (Thermo Fisher Scientific) and stored at −20°C. Cellular cholesterol was measured using the Amplex Red Cholesterol Assay Kit (Thermo Fisher Scientific) according to the manufacturer’s instructions. Briefly, the assay uses cholesterol esterase from *Pseudomonas* to hydrolyze cholesterol esters before cholesterol oxidase from *Streptomyces* oxidizes cellular cholesterol to yield hydrogen peroxide and cholestenone. The Amplex Red reagent reacts with hydrogen peroxide in the presence of horseradish peroxidase to produce fluorescent resorufin. Fluorescence was measured by a POLARstar Omega microplate reader using 530 nm excitation and 590 nm emission. Protein abundance was measured in samples using a DC protein assay (Bio-Rad, Hercules, CA, United States), and cholesterol concentrations were normalized to total cellular protein. The inter- and intra-assay coefficients of variation for the assay were < 6% and < 5% respectively.

### Immunofluorescence

To examine the distribution of actin filaments, cells were stained with Alexa Fluor 555-conjugated phalloidin (Thermo Fisher Scientific)^16^. Glass coverslips were seeded with 4 ×10^4^ cells per well in 24-well culture plates in complete medium for 24 h, before treatment in serum-free medium with vehicle, 10 µM dexamethasone, or 10 µM hydrocortisone for 24 h. The supernatants were discarded, and the cells were challenged with control or pyolysin for 2 h, or α-hemolysin for 24 h. At the end of the challenge period, cells were washed with PBS, fixed with 4% paraformaldehyde (Merck), washed in PBS, and then permeabilized in 0.2% Triton X-100. The cells were blocked for 30 min in PBS containing 0.5% bovine serum albumin and 0.1% Triton X-100, and then incubated for 1 h with Alexa Fluor 555 conjugated phalloidin (Thermo Fisher Scientific). Cells were washed with 0.1% Triton X-100 in PBS 3 times and mounted onto microscope slides using 40,6 diamidino-2-phenylindole to visualize cell nuclei (Vectashield with DAPI; Vector Laboratories Inc., Burlington, CA, USA). The cells were examined using an Axio Imager M1 fluorescence microscope and images captured using an AxioCamMR3 (Zeiss). The proportion of cells that had cytoskeletal changes (cytoskeletal contraction, disrupted shape, or loss of actin fiber definition) after pyolysin challenge were counted using > 135 cells per treatment across 3 independent images per replicate.

To further investigate changes in cell shape, cells were stained with CellMask Deep Red plasma membrane stain (Thermo Fisher Scientific). Briefly, 4 × 10^4^ HeLa cells per well were cultured on glass coverslips in 24-well culture plates in complete medium for 24 h, then treated in serum-free media containing vehicle or 10 µM dexamethasone for 24 h. Supernatants were discarded, and cells were challenged with control or pyolysin for 2 h. At the end of the challenge period, cells were washed with PBS, incubated with CellMask Deep Red for 10 min at 37°C, washed again with PBS, and fixed with 4% paraformaldehyde. Coverslips were mounted onto microscope slides using 40,6 diamidino-2-phenylindole to visualize cell nuclei, and the cells were examined using an Axio Imager M1 fluorescence microscope and images captured using an AxioCamMR3.

### Western blotting

At the end of experiments, supernatants were discarded, cells were washed with 300 μl ice-cold PBS, and lysed with 100 μl PhosphoSafe Extraction Reagent (Novagen, Darmstadt, Germany) for subsequent protein isolation and Western blotting^31^. Protein was extracted and quantified using a DC assay, and 10 μg/lane protein separated using 10% (vol/vol) SDS polyacrylamide gel electrophoresis. Proteins were then transferred onto a polyvinylidene difluoride membrane (GE Healthcare, Chalfont, St Giles, UK), and blocked for 1 h in Tris-buffered saline Tween 20 (TBST, Merck) with 5% BSA. The membranes were then incubated overnight at 4°C with primary antibodies diluted 1:1000 (or as specified) in blocking buffer. The primary antibodies were, diphosphorylated ERK1/2 (RRID: AB_477245; Merck), ERK1/2 (RRID: AB_2297336; Abcam, Cambridge, UK), phospho-p38 (RRID: AB_2139682, Cell Signaling, Danvers, MA, USA), p38 (RRID: AB_10999090, Cell Signaling), phospho-JNK (RRID:AB_331659; Cell Signaling), JNK (RRID: AB_2250373; Cell Signaling), α His pyolysin antibody^13^ (1:500 dilution), glucocorticoid receptor (RRID: AB_2631286, Cell Signaling), HMGCR (RRID: AB_2749818, Abcam, Cambridge, UK) or α-tubulin (RRID: AB_2210548; Cell Signaling). Membranes were then washed 5 times in TBST and incubated for 1 h at room temperature with 1:2500 dilution anti-rabbit IgG (RRID:AB_2099233; Cell Signalling) or anti-mouse IgG (RRID:AB_330924; Cell Signalling) in TBST with 5% BSA. Membranes were washed a further 5 times in TBST, and protein reactivity was visualized using enhanced chemiluminescence (Clarity Western ECL substrate, Bio-Rad). Membrane images (Supplementary Figure 10) were captured using a ChemiDoc XRS System (Bio-Rad), and the average peak density of bands was quantified and normalized to α-tubulin using Fiji^57^.

### Statistics and reproducibility

The statistical unit was each independent passage of cells. Data are reported as arithmetic mean ± s.e.m. with significance attributed when *P* < 0.05. Statistical analysis was performed using GraphPad Prism 9.0.1 (GraphPad Software, San Diego, California, USA) and SPSS ver 26.0 (IBM, Armonk, NY, USA). Comparisons between treatments were made using ANOVA, followed by post hoc tests for multiple comparisons as specified in Results. Proportions were compared using the Chi-square test.

### Reporting summary

Further information on research design is available in the Nature Portfolio Reporting Summary linked to this article.

## Supplementary information

Supplementary Information

Description of Additional Supplementary Files

Supplementary Data 1

Reporting summary

## Data Availability

All data generated or analyzed during this study, and uncropped Western blots, are included in this published article and its supplementary information files. The datasets generated and analyzed during the current study are available in Supplementary Data 1. All other data are available from the corresponding author on reasonable request.
