# Supplementary material for: Glucocorticoids increase tissue cell protection against pore-forming toxins from pathogenic bacteria
Source: Commun Biol. 2023 Feb 17;6:186. doi: 10.1038/s42003-023-04568-w (PMC9938277; doi:10.1038/s42003-023-04568-w)
Supplement: Supplementary file 1 — Supplementary Information [file 42003_2023_4568_MOESM1_ESM.pdf]

Glucocorticoids increase tissue cell protection against pore-forming toxins from pathogenic bacteria

Thomas J. R. Ormsby[1], Sian E. Owens[1], Matthew L. Turner[1], James G. Cronin[1], John J. Bromfield[2], I. Martin Sheldon[1]*

[1] Swansea University Medical School, Swansea University, Swansea SA2 8PP, UK.

[2] Department of Animal Sciences, University of Florida, Gainesville 32611, USA.

* Corresponding author: I. Martin Sheldon, Swansea University Medical School, Swansea University, Swansea SA2 8PP, UK. e-mail: i.m.sheldon@swansea.ac.uk

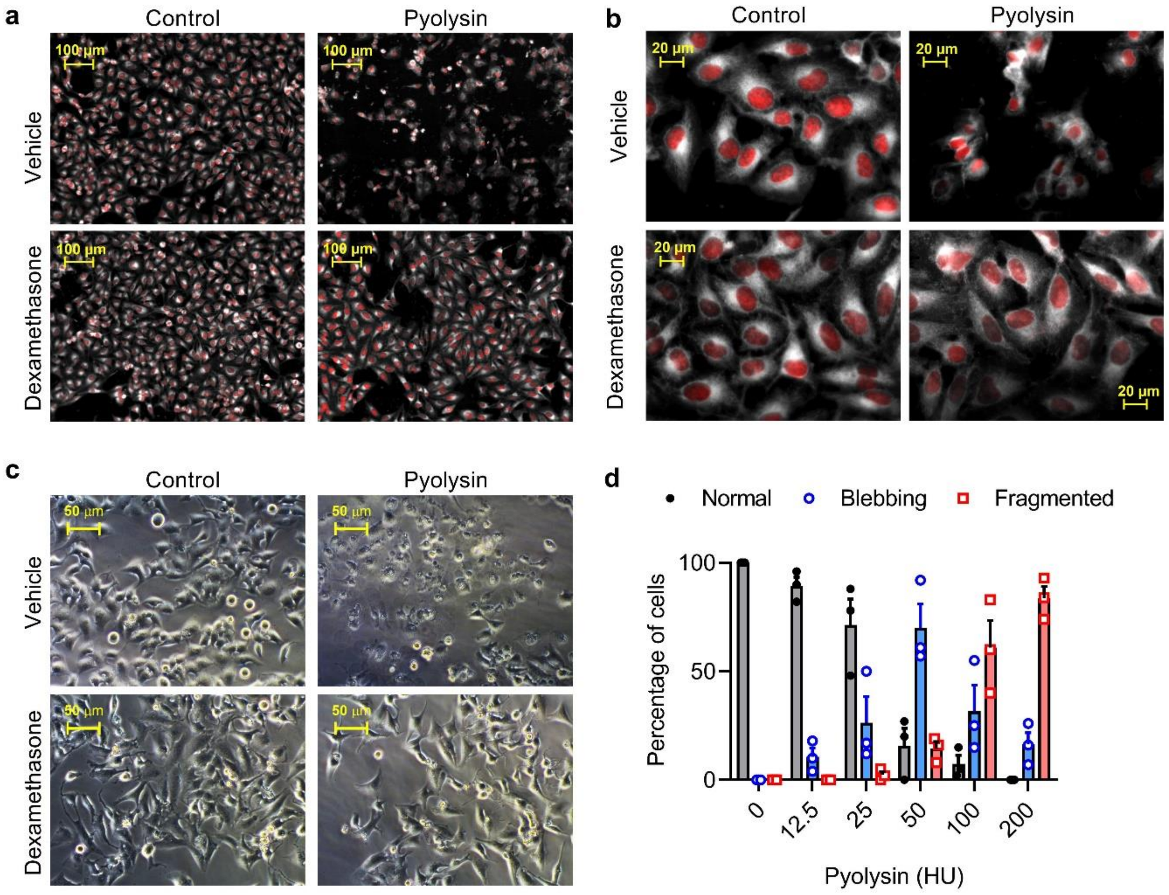

Supplementary Figure 1. Dexamethasone protects HeLa cells against pyolysin-induced changes in morphology

a–**c** HeLa cells were treated with vehicle or 10 µM dexamethasone for 24 h in serum-free medium, and then challenged with control or 100 HU/well pyolysin for 2 h. Cells were stained with CellMask plasma membrane stain to visualize plasma membranes (white) and fluorescent microscope images collected (nuclei are red, scale bars are **a** 100 µm and **b** 20 µm) or **c** transmitted light micrographs captured at the end of the experiment. Images are representative of 3 independent experiments. **d** Percentage of normal, blebbing, or fragmented HeLa cells cultured for 24 h in serum-free medium, then challenged with the indicated amount of pyolysin per well for 2 h. Data are mean ± s.e.m. from 4 independent experiments, with pyolysin causing cell blebbing and fragmentation (ANOVA, both $P < 0.001$).

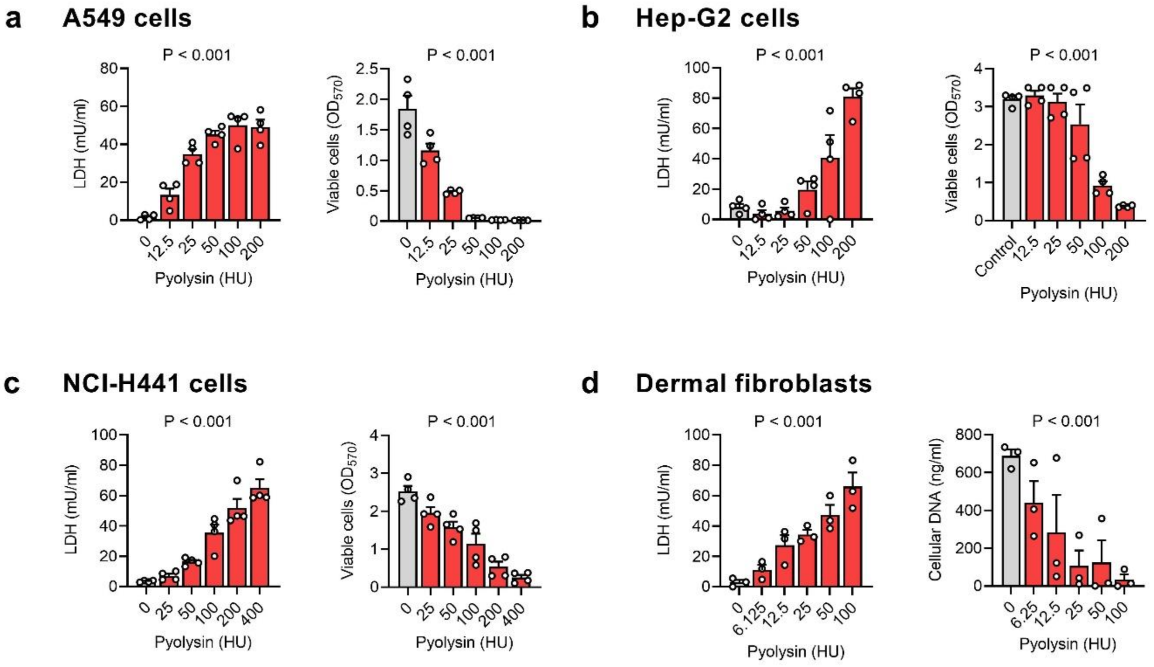

Supplementary Figure 2. Cytolytic activity of pyolysin

a A549 lung cells, **b** Hep-G2 liver cells, **c** NC1-H441 lung cells, and **d** primary normal human dermal fibroblasts were cultured for 24 h in serum-free medium and then challenged with the indicated amounts of pyolysin per well for 2 h. The leakage of LDH was measured in cell supernatants and cell viability was determined by MTT assay (**a**–**c**) or CyQUANT assay for cellular DNA (**d**). Data are mean + s.e.m. from 3 or 4 independent experiments; statistical significance was determined using one-way ANOVA and P-values reported.

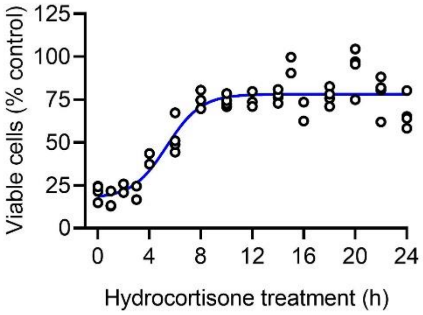

Supplementary Figure 3. Glucocorticoids protect cells against pyolysin

HeLa cells were treated with 10 µM hydrocortisone for the indicated times, challenged with control or 100 HU/well pyolysin for 2 h, and cell viability quantified by MTT assay. Pyolysin data are percentage of control challenge, with dots representing individual values across 4 independent experiments; the line is the least squares fit.

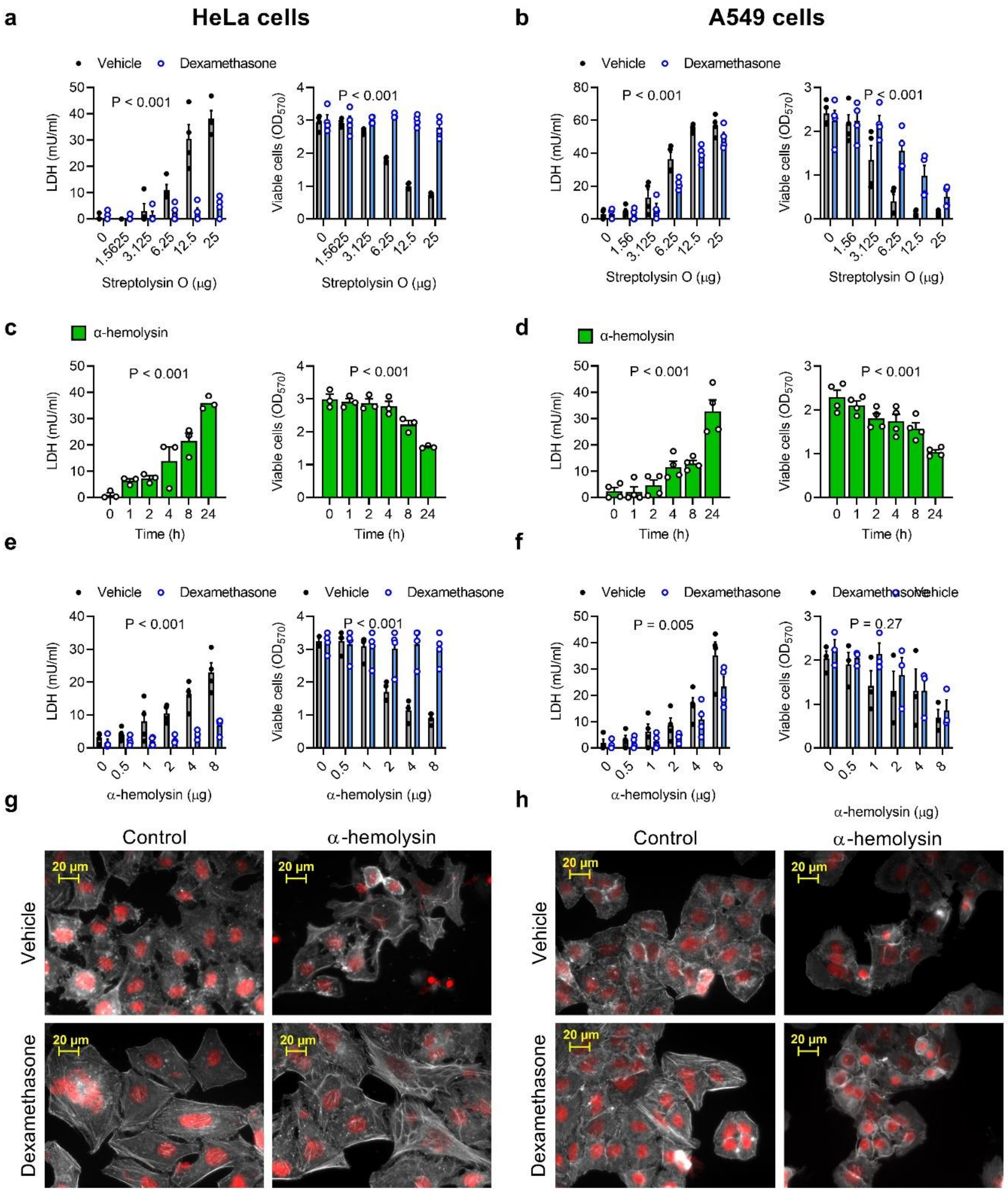

Supplementary Figure 4. Streptolysin O and α-hemolysin in HeLa and A549 cells

a HeLa cells or **b** A549 cells were treated with vehicle or 10 µM dexamethasone for 24 h, challenged with streptolysin O for 2 h, and LDH leakage and cell viability quantified. Data are mean ± s.e.m. from 4 independent experiments; statistical significance was determined using two-way ANOVA, and P-value reported for the effect of dexamethasone treatment. **c** HeLa and **d** A549 cells were cultured for 24 h in serum-free medium, and then challenged with control or 8 µg α-hemolysin for the indicated times. The leakage of LDH was measured in cell supernatants and cell viability was determined by MTT assay. Data are mean + s.e.m.

from 3 or 4 independent experiments; statistical significance was determined using one-way ANOVA and P-values reported. **e** HeLa cells or **f** A549 cells were treated with vehicle or 10 μM dexamethasone for 24 h, challenged with α-hemolysin for 24 h, and LDH leakage and cell viability quantified. Data are mean ± s.e.m. from 3 or 4 independent experiments; statistical significance was determined using two-way ANOVA, and P-value reported for the effect of dexamethasone treatment. **g** Fluorescent microscope images of HeLa cells or **f** A549 cells treated with vehicle or 10 μM dexamethasone for 24 h, and then challenged with control or 8 μg α-hemolysin for 24 h, and stained with fluorescent phalloidin (white) and DAPI (red); images are representative of 3 independent experiments.

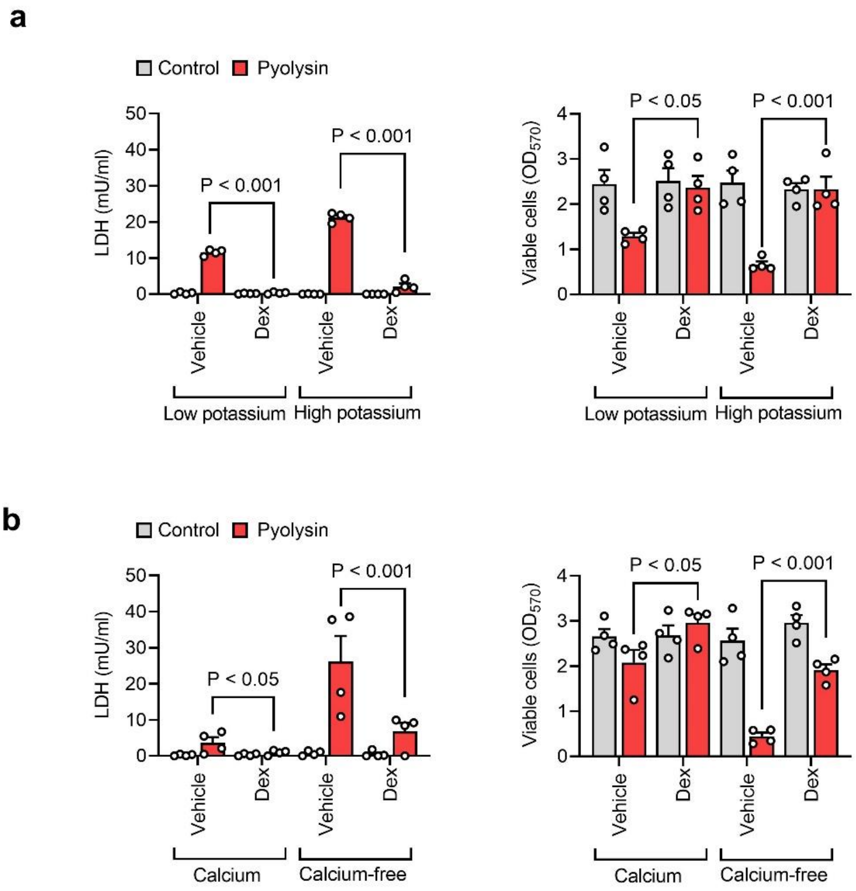

Supplementary Figure 5. Potassium and calcium does not affect dexamethasone cytoprotection against pyolysin

a Hela cells were treated with control serum-free medium containing vehicle or 10 µM dexamethasone for 24 h, and then challenged with control or 100 HU/well pyolysin for 2 h in low-potassium or high-potassium buffer. The leakage of LDH was measured in cell supernatants and viable cells assessed by MTT assay. Data are mean + s.e.m. from 4 independent experiments; statistical significance was determined using two-way ANOVA and Bonferroni multiple comparison test. **b** Hela cells were treated with control serum-free medium containing vehicle or 10 µM dexamethasone for 24 h, and then challenged with control or 100 HU/well pyolysin for 2 h in in calcium or calcium-free conditions for 2 h. The leakage of LDH was measured in cell supernatants and viable cells assessed by MTT assay. Data are mean + s.e.m. with dots representing the values of cells from 4 independent passages; statistical significance was determined using two-way ANOVA with Bonferroni multiple comparison test.

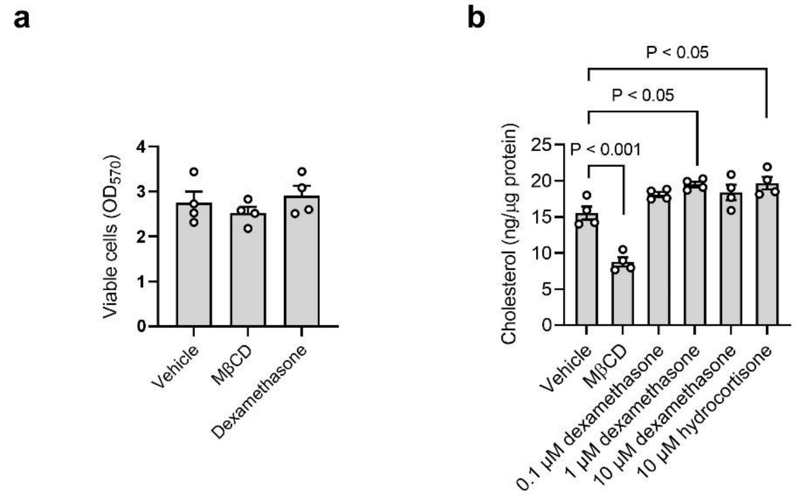

Supplementary Figure 6. Glucocorticoids and cellular cholesterol

a HeLa cells in serum-free medium were treated with vehicle, 1 mM methyl-β-cyclodextrin (MβCD) or 10 µM dexamethasone for 24 h and cell viability quantified by MTT assay. **b** A549 cells in serum-free medium were treated with vehicle, 1 mM methyl-β-cyclodextrin (MβCD), 0.1, 1 or 10 µM dexamethasone or 10 µM hydrocortisone for 24 h. Total cellular cholesterol was quantified and normalized to total cellular protein. Data are mean + s.e.m. from 4 independent experiments; statistical significance was determined using one-way ANOVA with Dunnett's post hoc test.

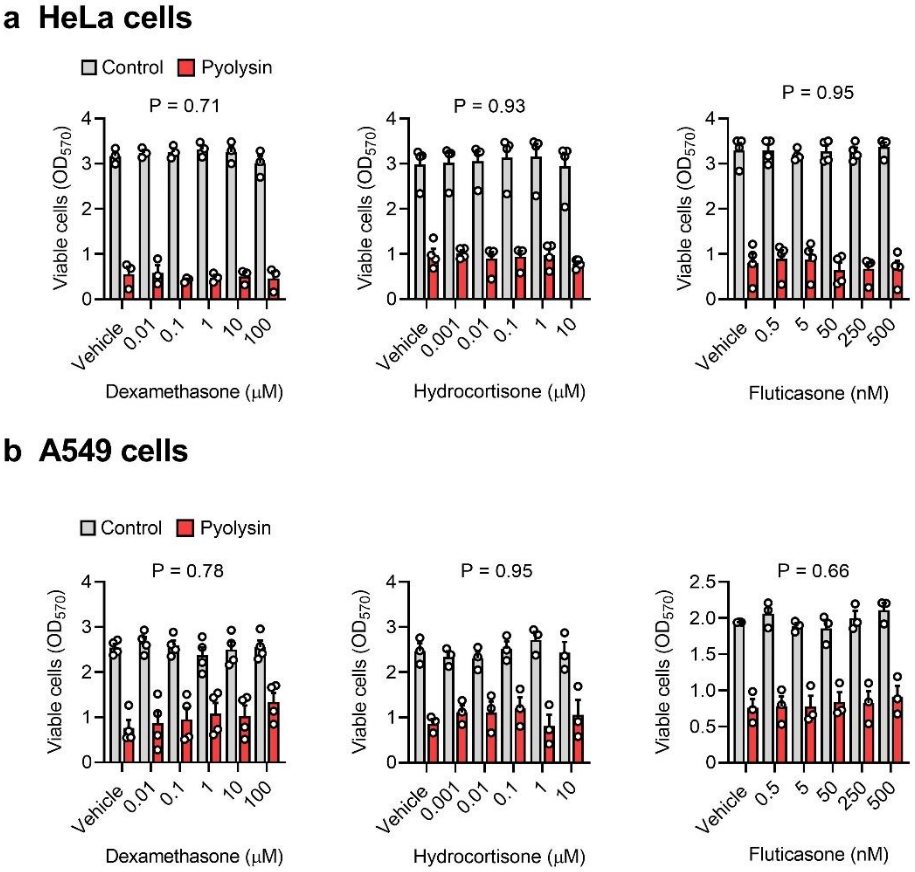

Supplementary Figure 7. Serum inhibits glucocorticoid cytoprotection in A549 cells

a HeLa cells or **b** A549 cells were cultured in medium containing 10% serum and treated with vehicle, dexamethasone, hydrocortisone, or fluticasone propionate for 24 h, then challenged with control or 100 HU/well pyolysin for 2 h, and viable cells determined by MTT assay. Data are mean + s.e.m. from 3 or 4 independent experiments; statistical significance was determined using two-way ANOVA and P-values reported for the dexamethasone effect on the pyolysin challenge.

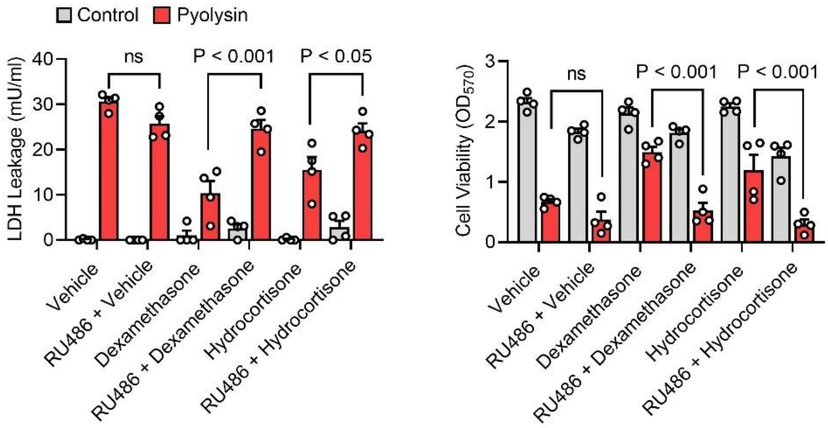

Supplementary Figure 8. Glucocorticoid cytoprotection is dependent on the glucocorticoid receptor in A549 cells

a A549 cells were cultured in medium with or without 10 µM RU486, and treated with vehicle, 10 µM dexamethasone or 10 µM hydrocortisone for 24 h, and then challenged with control or 25 HU/well pyolysin for 2 h, and the leakage of LDH was measured in cell supernatants and viable cells determined by MTT assay. Data are mean + s.e.m. from 4 independent experiments; statistical significance was determined using ANOVA and Tukey's post hoc test, ns = not significant.

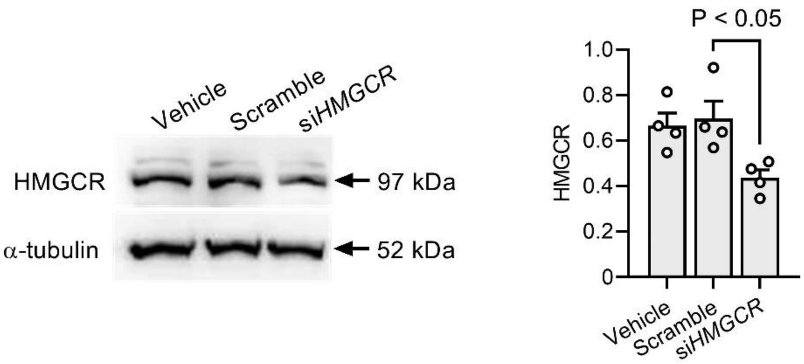

Supplementary Figure 9. Targeting HeLa cell HMGCR

Representative Western blot of HMGCR and α-tubulin for HeLa cells transfected with scrambled siRNA or siRNA targeting *HMGCR*; images representative of 4 independent experiments, with densitometry data normalized to α-tubulin and presented as mean + s.e.m.; statistical significance was determined using ANOVA with Tukey post hoc test.

ERK Western blots

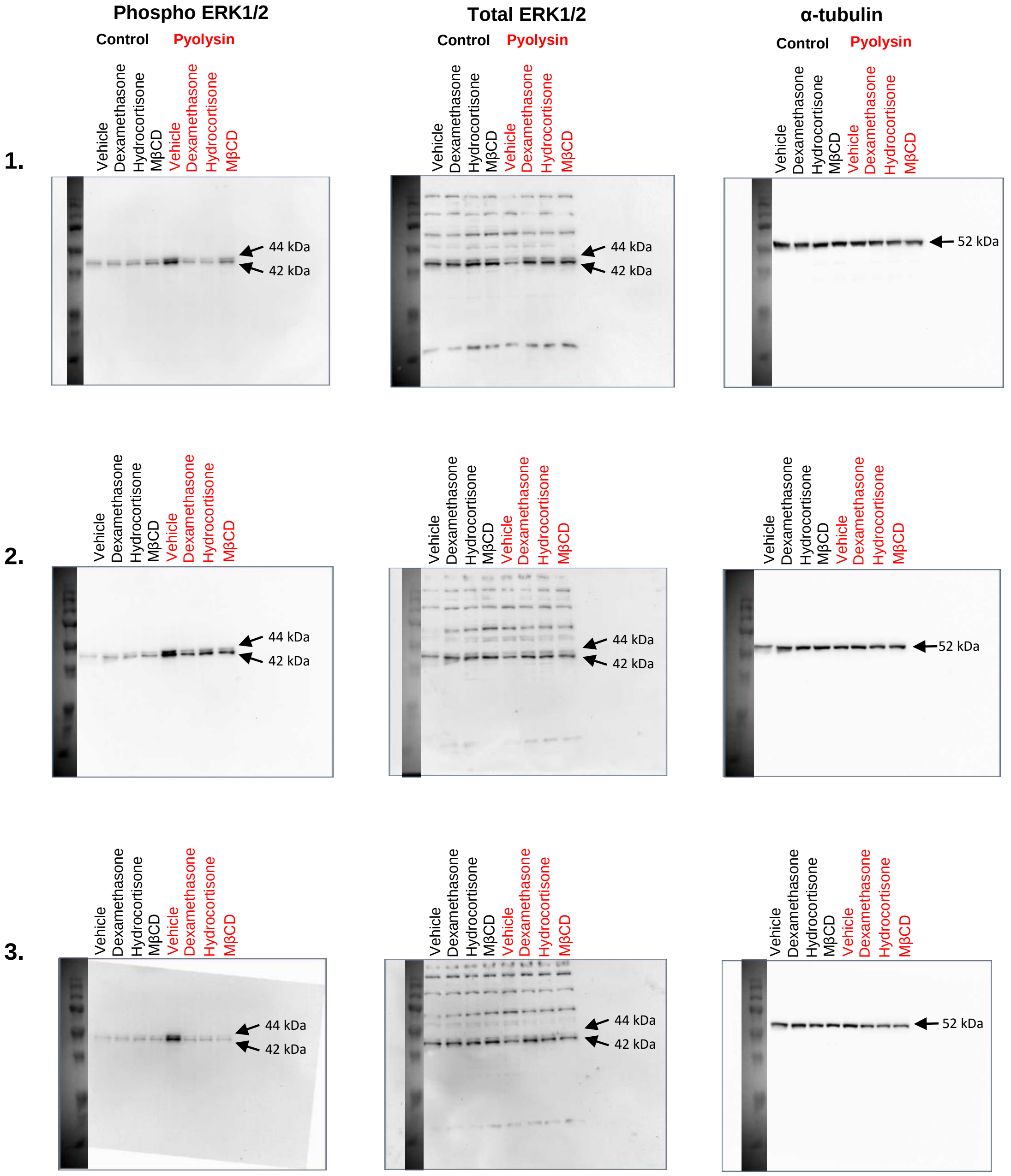

p38 Western blots

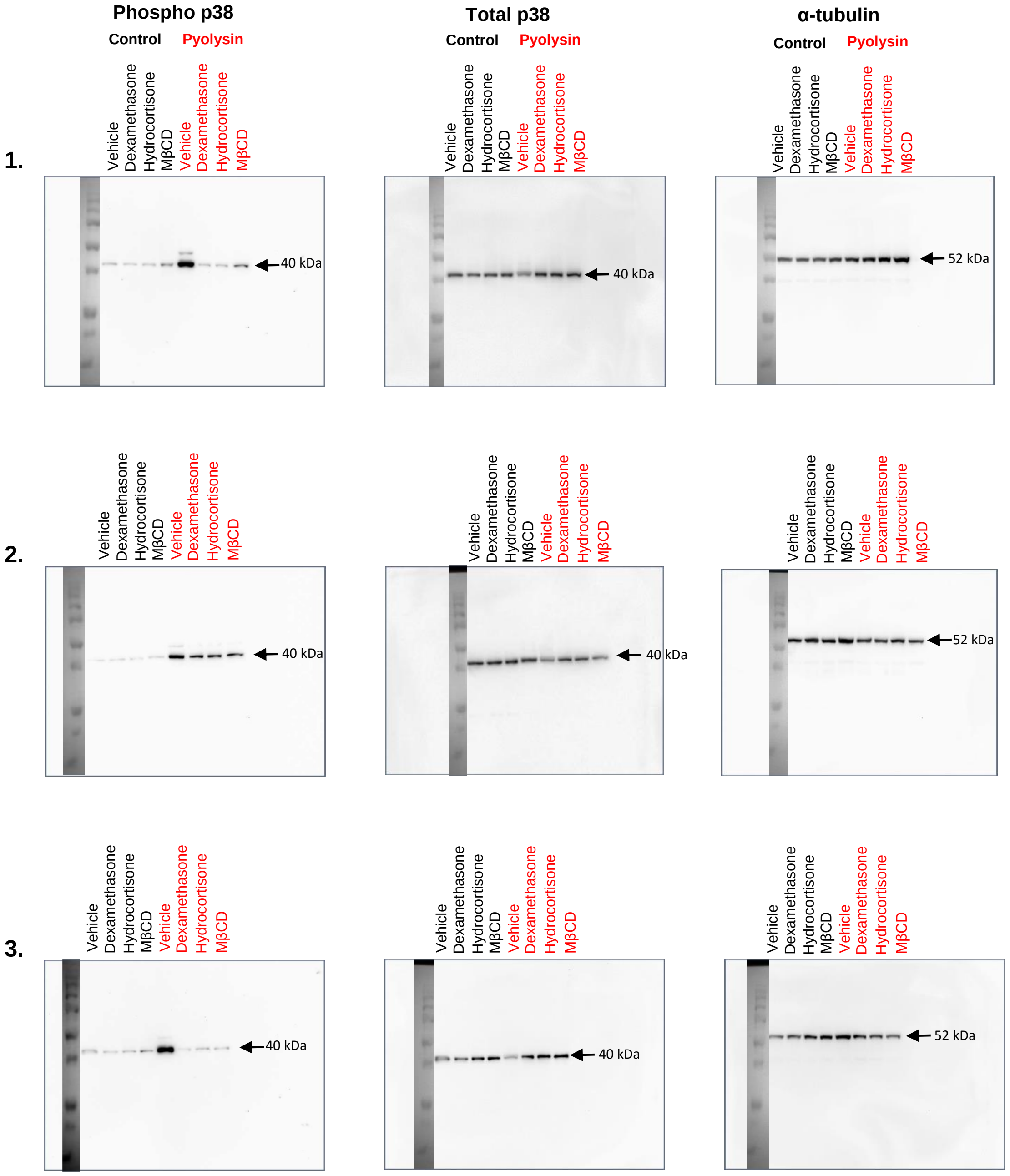

JNK Western blots

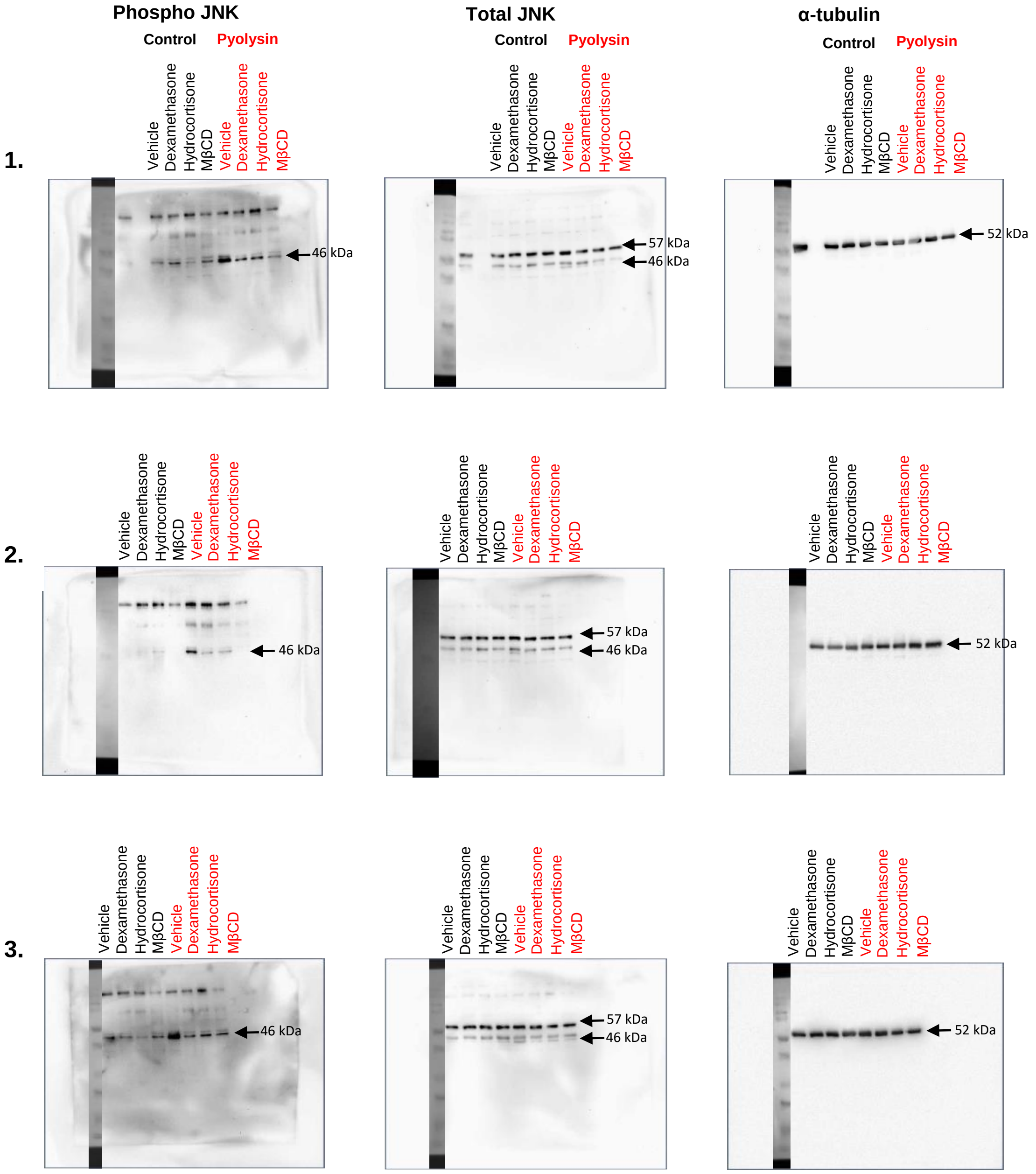

Pyolysin Western blots for HeLa cells

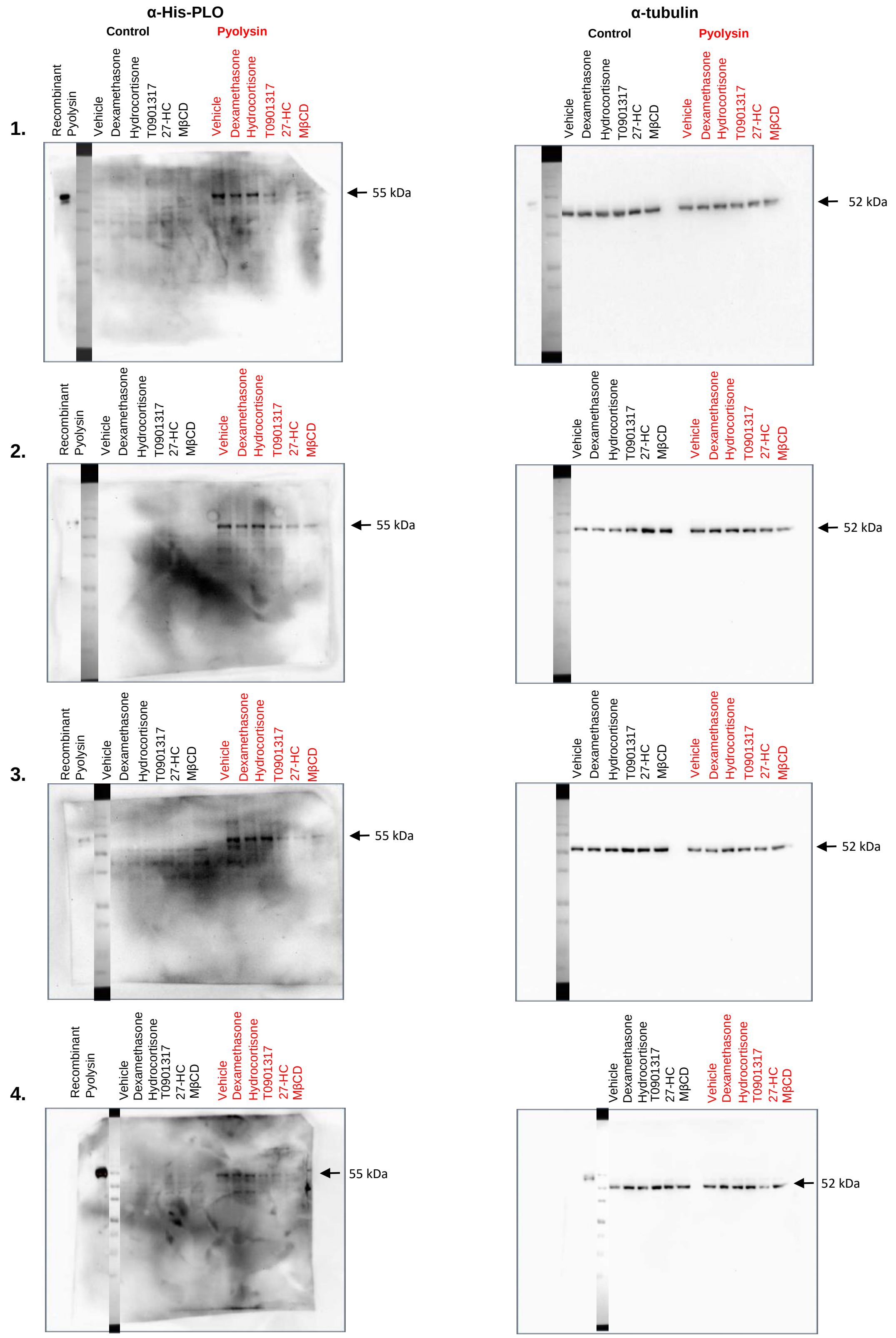

Glucocorticoid receptor Western blots

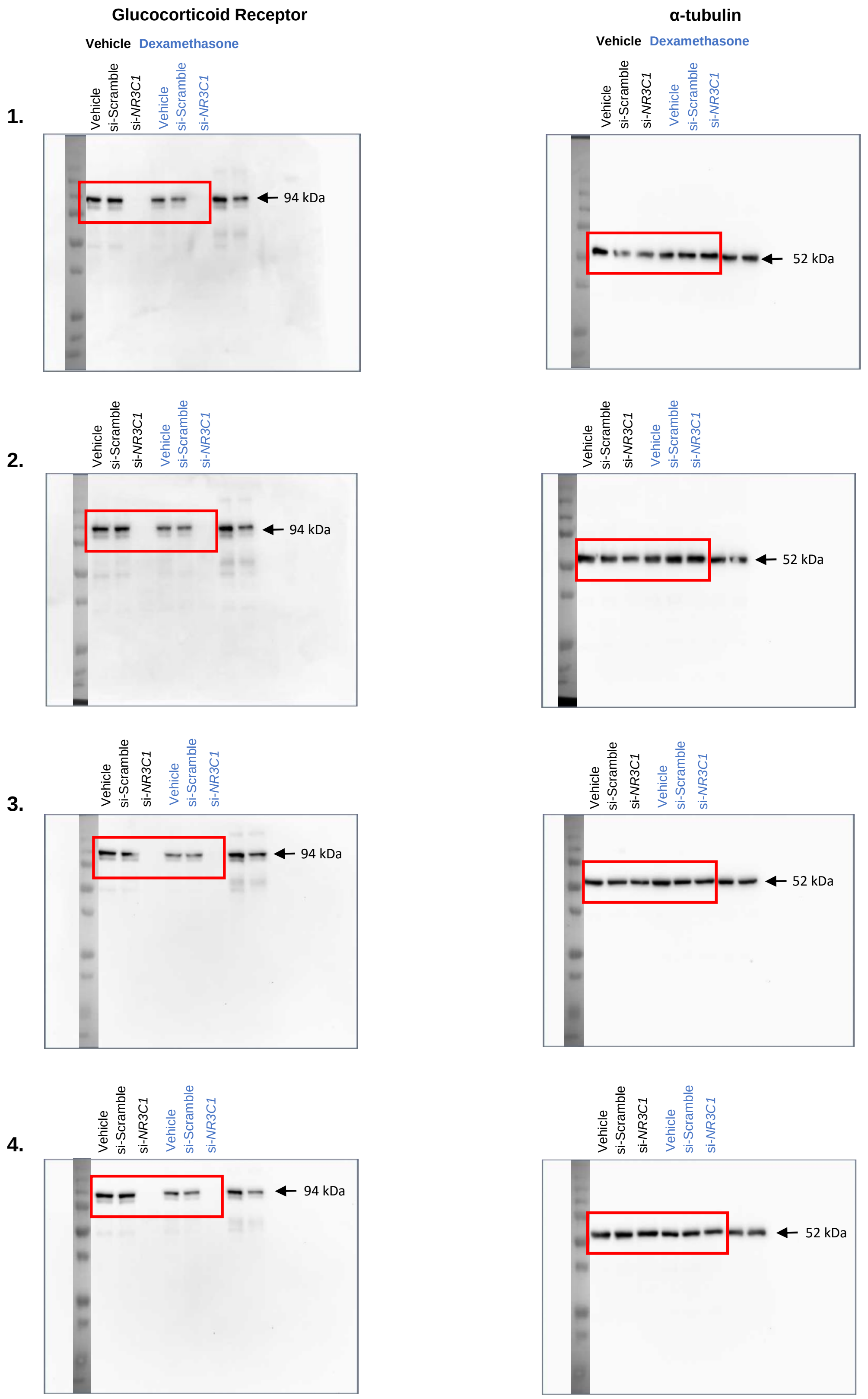

HMGCR Western blots

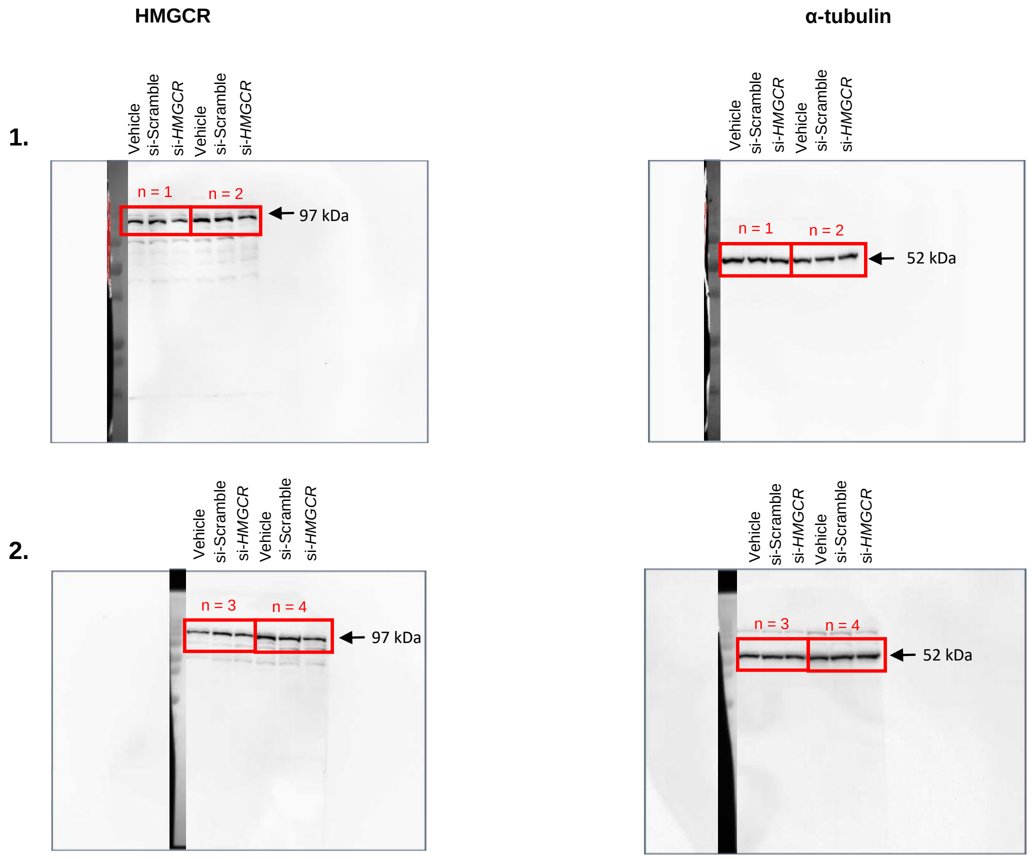

Supplementary Figure 10. Uncropped Western blots

Uncropped Western blots used in Figure 5 (phosphor and total ERK, p38 and JNK), Figure 6c (pyolysin, α-His-PLO), Figure 8c (glucocorticoid receptor, GR), and Supplementary Figure 9 (HMGCR), with α-tublin loading controls.

Supplemental Table 1. Genes regulated by dexamethasone or serum.

Changes in gene expression associated with dexamethasone (Dex) or serum, with differentially regulated genes highlighted (see key). Gene abbreviation and HUGO Gene Nomenclature Committee identity (HGNC ID) are indicated.

Inflammation

Gene	HGNC ID	Dex	Serum
ALOX5AP	436	↑ [1]	
CCL2	10618	↓ [1, 2]	↓ [3]
CCL20	10619	↑ [1]	
CXCL12	10672		↑ [3]
CXCL2	4603		↓ [3]
CXCL8	6025	↓ [4]	↑ [5]
ICAM1	5344	↓ [6]	↑ [3]
IL11	5966	↓ [1]	
IL1B	5992	↓ [7]	↓ [3]
PDE4B	8781	↓ [1]	
PTGS2	9605	↓ [1]	↑ [3]
THBD	11784	↑ [1]	
TSC22D3	3051	↑ [1]	

Metabolism

Gene	HGNC ID	Dex	Serum
ANGPTL4	16039	↑ [1]	
B3GNT5	15684	↑ [1]	
ETNK2	25575	↑ [1]	
MGAM	7043	↑ [1]	
TXNIP	16952	↑ [8]	↓ [3]

Cholesterol Biosynthesis

Gene	HGNC ID	Dex	Serum
CYP51A1	2649		↓ [3]
FDFT1	3629		↓ [3]
HMGCR	5006	↑ [9]	↓ [3, 9]
IDI1	5387		↓ [3]
SQLE	11279		↓ [3]

Transport

Gene	HGNC ID	Dex	Sreum
MT1A	7393	↑ [10]	↑ [3]
MT1B	7394		↑ [3]
MT1IP	7401	↑ [1]	↑ [3]
SCNN1A	10599	↑ [1]	
SLC19A2	10938	↑ [1]	
SLC26A2	10994	↑ [1]	
STOM	2282	↑ [1]	

Growth and Apoptosis

Gene	HGNC ID	Dex	Serum
BIRC3	591	↑ [1]	
CCNA2	1578		↑ [3]
CCNB1	1579		↑ [3]
CCND1	1582		↑ [3]
CDK1	1722		↑ [3]
CDK7	1788		↑ [3]
CDKN1C	1786	↑ [1]	↓ [3]
CENPF	1857		↑ [3]
CKS2	2000		↑ [3]
CTPS1	2519		↑ [3]
CUL1	2551	↓ [1]	
FGFBP1	19695	↓ [1]	
GADD45B	4096	↑ [1]	
ID2	5361		↑ [3]
ID3	5362		↑ [3]
LBR	6518		↓ [3]
MAD2L1	6763		↑ [3]
MFGE8	7036	↑ [1]	
MKI67	7101		↑ [3]
PCNA	8729		↑ [3]
RRM1	10451		↑ [3]
RRM2	10452		↑ [3]
S100P	10504	↑ [1]	
SERPINB9	8955	↓ [1]	
SERTAD2	30784	↓ [1]	
SIAH1	10857		↓ [3]
SNAI2	11094	↑ [1]	
SPRY1	11269	↑ [1]	
TNFAIP3	11896	↑ [1]	
TOP2A	11989		↑ [3]
WEE1	12761		↓ [3]

Key	
↑	Induced
↓	Repressed
	Differentially regulated
	Similarly regulated

Cell Signalling

Gene	HGNC ID	Dex	Serum
ACKR3	23692	↓ [1]	
AKAP13	371	↑ [1]	
ANKRD1	15819	↑ [1]	
ARL8	25192	↓ [1]	
BHLHE40	1046	↓ [1]	
CDC42EP3	16943	↑ [1]	
CPEB4	21747	↑ [1]	
DNER	24456	↑ [1]	
DUS1	3064	↑ [10]	↑ [3]
EDN2	3177	↑ [1]	
ENC1	3345	↓ [1]	
EPB41L4B	19818	↑ [1]	
FGD4	19125	↑ [1]	
FKBP5	3721	↑ [1, 15]	↓ [15]
GEM	4234	↓ [1]	↓ [3]
IHPK3	17269	↑ [1]	
IRS2	6126	↑ [1]	
KIT	6342		↓ [3]
PLK2	19699	↓ [1]	↑ [3]
POU5F1	9221	↑ [1]	
PPP1R14C	14952	↑ [1]	
RASD1	15828	↑ [17]	↓ [18]
RGS2	9998	↑ [1]	
ROR1	10256	↑ [19]	↓ [3]
S1PR1	3165		↑ [3]
SEC14L1	10698	↑ [1]	
SGK1	10810	↑ [20]	↓ [3]
TGFBR3	11774	↑ [1]	↓ [3]
ZIC2	12873	↓ [1]	

Other

Gene	HGNC ID	Dex	Serum
ABHD2	18717	↑ [1]	
ADGRF4	19011	↑ [1]	
AMIGO2	24073	↓ [1]	
ARRDC3	29263	↓ [1]	
CALD1	1441	↑ [11, 12]	↓ [3]
CAVIN2	10690	↑ [1]	↑ [13]
CDH2	1759		↑ [3]
CFHR1	4888		↓ [3]
COL1A1	2197		↓ [3]
DNAJC15	20325	↑ [1]	
FBN2	3604		↓ [3]
FGF2	3676	↑ [14]	↑ [3]
FGF3	3681		↓ [3]
FGF7	3685	↓ [16]	↑ [3]
FHL2	3703		↑ [3]
FLVCR2	20105	↑ [1]	
FURIN	8568		↑ [3]
GPR153	23618	↑ [1]	
HKDC1	23302	↓ [1]	
KTN1	6467	↓ [1]	
LAMA2	6482		↓ [3]
LRRC8A	19027	↑ [1]	
MME	7154		↓ [3]
NAV3	15998	↓ [1]	
OTULINL	25629	↑ [1]	
PLEKHA7	27049	↑ [1]	
PLOD2	9082		↑ [3]
PMP2	9117	↓ [1]	
PNRC1	17278		↓ [3]
PRRG4	30799	↑ [1]	
SERPINB2	8584		↑ [3]
SERPINE1	8583	↑ [11]	↑ [3]
SPINK5L3	27200	↑ [1]	
SPTBN1	11275		↓ [3]
SRGN	9361	↑ [1]	
TFPI2	11761	↓ [11]	↑ [3]
TNS4	24352	↑ [1]	

Key	
↑	Induced
↓	Repressed
(yellow)	Differentially regulated
(grey)	Similarly regulated

Supplementary References

1. Wang J-C, Derynck MK, Nonaka DF, Khodabakhsh DB, Haqq C, Yamamoto KR. Chromatin immunoprecipitation (ChIP) scanning identifies primary glucocorticoid receptor target genes. *Proceedings of the National Academy of Sciences* **101**, 15603-15608 (2004).
2. Dhawan L, Liu B, Blaxall BC, Taubman MB. A novel role for the glucocorticoid receptor in the regulation of monocyte chemoattractant protein-1 mRNA stability. *J Biol Chem* **282**, 10146-10152 (2007).
3. Iyer VR*, et al.* The transcriptional program in the response of human fibroblasts to serum. *Science* **283**, 83-87 (1999).
4. Chang MM, Juarez M, Hyde DM, Wu R. Mechanism of dexamethasone-mediated interleukin-8 gene suppression in cultured airway epithelial cells. *Am J Physiol Lung Cell Mol Physiol* **280**, L107-115 (2001).
5. Kim SW, Kim SJ, Langley RR, Fidler IJ. Modulation of the cancer cell transcriptome by culture media formulations and cell density. *Int J Oncol* **46**, 2067-2075 (2015).
6. van de Stolpe A, Caldenhoven E, Raaijmakers JA, van der Saag PT, Koenderman L. Glucocorticoid-mediated repression of intercellular adhesion molecule-1 expression in human monocytic and bronchial epithelial cell lines. *Am J Respir Cell Mol Biol* **8**, 340-347 (1993).
7. Amano Y, Lee SW, Allison AC. Inhibition by glucocorticoids of the formation of interleukin-1 alpha, interleukin-1 beta, and interleukin-6: mediation by decreased mRNA stability. *Mol Pharmacol* **43**, 176-182 (1993).
8. Wang Z, Rong YP, Malone MH, Davis MC, Zhong F, Distelhorst CW. Thioredoxin-interacting protein (txnip) is a glucocorticoid-regulated primary response gene involved in mediating glucocorticoid-induced apoptosis. *Oncogene* **25**, 1903-1913 (2006).
9. Cavenee WK, Melnykovych G. Induction of 3-hydroxy-3-methylglutaryl coenzyme A reductase in HeLa cells by glucocorticoids. *J Biol Chem* **252**, 3272-3276 (1977).
10. Lasa M, Abraham SM, Boucheron C, Saklatvala J, Clark AR. Dexamethasone causes sustained expression of mitogen-activated protein kinase (MAPK) phosphatase 1 and phosphatase-mediated inhibition of MAPK p38. *Mol Cell Biol* **22**, 7802-7811 (2002).
11. Mostafa MM*, et al.* Glucocorticoid-driven transcriptomes in human airway epithelial cells: commonalities, differences and functional insight from cell lines and primary cells. *BMC Med Genomics* **12**, 29 (2019).
12. Mayanagi T, Morita T, Hayashi K, Fukumoto K, Sobue K. Glucocorticoid receptor-mediated expression of caldesmon regulates cell migration via the reorganization of the actin cytoskeleton. *J Biol Chem* **283**, 31183-31196 (2008).
13. Breen MR, Camps M, Carvalho-Simoes F, Zorzano A, Pilch PF. Cholesterol depletion in adipocytes causes caveolae collapse concomitant with proteosomal degradation of cavin-2 in a switch-like fashion. *PLoS One* **7**, e34516 (2012).
14. Meisinger C, Zeschnigk C, Grothe C. In vivo and in vitro effect of glucocorticoids on fibroblast growth factor (FGF)-2 and FGF receptor 1 expression. *J Biol Chem* **271**, 16520-16525 (1996).

15. Yang N*, et al.* Serum cholesterol selectively regulates glucocorticoid sensitivity through activation of JNK. *J Endocrinol* **223**, 155-166 (2014).
16. Chedid M, Hoyle JR, Csaky KG, Rubin JS. Glucocorticoids inhibit keratinocyte growth factor production in primary dermal fibroblasts. *Endocrinology* **137**, 2232-2237 (1996).
17. Kemppainen RJ, Behrend EN. Dexamethasone rapidly induces a novel ras superfamily member-related gene in AtT-20 cells. *J Biol Chem* **273**, 3129-3131 (1998).
18. Wang L, Mitsui T, Ishida M, Izawa M, Arita J. Rasd1 is an estrogen-responsive immediate early gene and modulates expression of late genes in rat anterior pituitary cells. *Endocr J* **64**, 1063-1071 (2017).
19. Obradović MMS*, et al.* Glucocorticoids promote breast cancer metastasis. *Nature* **567**, 540-544 (2019).
20. Wang D, Zhang H, Lang F, Yun CC. Acute activation of NHE3 by dexamethasone correlates with activation of SGK1 and requires a functional glucocorticoid receptor. *Am J Physiol Cell Physiol* **292**, C396-404 (2007).
