# Supplementary material for: Glucocorticoids increase tissue cell protection against pore-forming toxins from pathogenic bacteria
Source: Commun Biol. 2023 Feb 17;6:186. doi: 10.1038/s42003-023-04568-w (PMC9938277; doi:10.1038/s42003-023-04568-w)
Supplement: Supplementary file 4 — Reporting summary [file 42003_2023_4568_MOESM4_ESM.pdf]

nature portfolio

Corresponding author(s): Iain Martin Sheldon

Last updated by author(s): Jan 31, 2023

Reporting Summary

Nature Portfolio wishes to improve the reproducibility of the work that we publish. This form provides structure for consistency and transparency in reporting. For further information on Nature Portfolio policies, see our Editorial Policies and the Editorial Policy Checklist.

Statistics

For all statistical analyses, confirm that the following items are present in the figure legend, table legend, main text, or Methods section.

n/a	Confirmed	
☐	☒	The exact sample size (*n*) for each experimental group/condition, given as a discrete number and unit of measurement
☐	☒	A statement on whether measurements were taken from distinct samples or whether the same sample was measured repeatedly
☐	☒	The statistical test(s) used AND whether they are one- or two-sided<br>*Only common tests should be described solely by name; describe more complex techniques in the Methods section.*
☐	☒	A description of all covariates tested
☐	☒	A description of any assumptions or corrections, such as tests of normality and adjustment for multiple comparisons
☐	☒	A full description of the statistical parameters including central tendency (e.g. means) or other basic estimates (e.g. regression coefficient) AND variation (e.g. standard deviation) or associated estimates of uncertainty (e.g. confidence intervals)
☐	☒	For null hypothesis testing, the test statistic (e.g. *F*, *t*, *r*) with confidence intervals, effect sizes, degrees of freedom and *P* value noted<br>*Give P values as exact values whenever suitable.*
☒	☐	For Bayesian analysis, information on the choice of priors and Markov chain Monte Carlo settings
☒	☐	For hierarchical and complex designs, identification of the appropriate level for tests and full reporting of outcomes
☐	☒	Estimates of effect sizes (e.g. Cohen's *d*, Pearson's *r*), indicating how they were calculated

Our web collection on statistics for biologists contains articles on many of the points above.

Software and code

Policy information about availability of computer code

Data collection	Membrane images were captured using a ChemiDoc XRS System (Bio-Rad), and the average peak density of bands was quantified and normalized to α tubulin using Fiji (Schindelin, J., Arganda-Carreras, I., Frise, E., Kaynig, V., Longair, M., Pietzsch, T., … Cardona, A. (2012). Fiji: an open-source platform for biological-image analysis. Nature Methods, 9(7), 676–682. doi:10.1038/nmeth.2019)
Data analysis	GraphPad Prism 9.0.1 (GraphPad Software, San Diego, California, USA)<br>SPSS ver 26.0 (IBM, Armonk, NY, USA)

For manuscripts utilizing custom algorithms or software that are central to the research but not yet described in published literature, software must be made available to editors and reviewers. We strongly encourage code deposition in a community repository (e.g. GitHub). See the Nature Portfolio guidelines for submitting code & software for further information.

Data

Policy information about availability of data

All manuscripts must include a data availability statement. This statement should provide the following information, where applicable:

- Accession codes, unique identifiers, or web links for publicly available datasets
- A description of any restrictions on data availability
- For clinical datasets or third party data, please ensure that the statement adheres to our policy

All data generated or analyzed during this study are included in this published article and its supplementary information files.

Human research participants

Policy information about studies involving human research participants and Sex and Gender in Research.

Reporting on sex and gender	Adult primary human chondrocytes were isolated from waste nasoseptal surgical tissue collected with informed consent from healthy donors undergoing septorhinoplasty at Singleton and Morriston Hospitals, Swansea, UK. Age and gender of 3 donors were not disclosed to researchers.
Population characteristics	See above.
Recruitment	Adult primary human chondrocytes were isolated from waste nasoseptal surgical tissue collected with informed consent from healthy donors undergoing septorhinoplasty at Singleton and Morriston Hospitals, Swansea, UK.
Ethics oversight	Adult primary human chondrocytes were isolated from waste nasoseptal surgical tissue collected with informed consent from healthy donors undergoing septorhinoplasty at Singleton and Morriston Hospitals, Swansea, UK, with approval from the Swansea Bay University Health Board (IRAS ID 99202).

Note that full information on the approval of the study protocol must also be provided in the manuscript.

Field-specific reporting

Please select the one below that is the best fit for your research. If you are not sure, read the appropriate sections before making your selection.

☒ Life sciences ☐ Behavioural & social sciences ☐ Ecological, evolutionary & environmental sciences

For a reference copy of the document with all sections, see nature.com/documents/nr-reporting-summary-flat.pdf

Life sciences study design

All studies must disclose on these points even when the disclosure is negative.

Sample size	Sample size was based on our previous published work that showed sufficient power to detect meaningful differences, and using statistical power calculations.
Data exclusions	No data was excluded from the study.
Replication	Main effects of glucocorticoid treatment on protection against pore-forming toxins was consistently replicated in at least 3 independent experiments for each of eight different cell types and using three different pore-forming toxins, as reported in the manuscript.
Randomization	Each in vitro experiment was performed using independent cell passages on independent days but there was no formal randomization.
Blinding	Investigators were not blinded.

Reporting for specific materials, systems and methods

We require information from authors about some types of materials, experimental systems and methods used in many studies. Here, indicate whether each material, system or method listed is relevant to your study. If you are not sure if a list item applies to your research, read the appropriate section before selecting a response.

Materials & experimental systems

n/a	Involved in the study	
☐	☒	Antibodies
☐	☒	Eukaryotic cell lines
☒	☐	Palaeontology and archaeology
☒	☐	Animals and other organisms
☒	☐	Clinical data
☒	☐	Dual use research of concern

Methods

n/a	Involved in the study	
☒	☐	ChIP-seq
☒	☐	Flow cytometry
☒	☐	MRI-based neuroimaging

Antibodies

Antibodies used	diphosphorylated ERK1/2 (RRID: AB_477245; Merck)

Antibodies used

ERK1/2 (RRID: AB_2297336; Abcam, Cambridge, UK)
phospho-p38 (RRID: AB_2139682, Cell Signaling, Danvers, MA, USA)
p38 (RRID: AB_10999090, Cell Signaling)
phospho-JNK (RRID:AB_331659; Cell Signaling)
JNK (RRID: AB_2250373; Cell Signaling)
α His pyolysin antibody (generously gifted by Prof. B.H. Jost, University of Arizona, USA 1:500 dilution; as described in Billington SJ, Jost BH, Cuevas WA, Bright KR, Songer JG. The Arcanobacterium (Actinomyces) pyogenes hemolysin, pyolysin, is a novel member of the thiol-activated cytolysin family. J Bacteriol 179, 6100-6106 (1997).
glucocorticoid receptor (RRID: AB_2631286, Cell Signaling)
HMGCR (RRID: AB_2749818, Abcam, Cambridge, UK)
α tubulin (RRID: AB_2210548; Cell Signaling)
anti-rabbit IgG (RRID:AB_2099233; Cell Signalling)
anti-mouse IgG (RRID:AB_330924; Cell Signalling)

Validation

Antibodies were chosen based on the validation statements for species (human) and applications (WB) on the manufacturers' websites, appropriate molecular size on Western blots, and provision of Research Resource Identifiers (RRIDs); except for α His pyolysin antibody (generously gifted by Prof. B.H. Jost, University of Arizona, USA 1:500 dilution; as described in Billington SJ, et al 1997 The Arcanobacterium (Actinomyces) pyogenes hemolysin, pyolysin, is a novel member of the thiol-activated cytolysin family. J Bacteriol 179, 6100-6106), which was also validated in Amos MR, et al. 2014 Differential endometrial cell sensitivity to a cholesterol-dependent cytolysin links Trueperella pyogenes to uterine disease in cattle. Biol Reprod 90, 54.

Eukaryotic cell lines

Policy information about cell lines and Sex and Gender in Research

Cell line source(s)

Human cervical epithelial HeLa cells (CCL2, 93021013; European Collection of Authenticated Cell Cultures (ECACC), Public Health England, Salisbury, UK)
Human lung epithelial A549 cells (CCL-185; ATCC, Middlesex, UK)
Human liver epithelial HepG2 cells (HB-8065; ATCC)
Human lung epithelial NCI-H441 cells (HTB-174; ATCC)
Primary normal human dermal fibroblasts (C-12302; Promocell, Heidelberg, Germany)
Primary normal human lung fibroblasts were obtained from normal healthy human lung parenchyma (HLF, PCS-201-013; ATCC).
Normal human prostate PNT2 cells (95012613; ECACC)
Normal human mesothelial MET5A cells (CRL-9444; ATCC)
Primary human chondrocytes were isolated from nasoseptal cartilage tissue, which was sequentially digested with 0.2% (w/v) pronase (Merck) for 40 min followed by 0.24% (w/v) collagenase solution (Merck) for 16 h, as described previously (Oseni AO et al 2013 J Surg Res 181, 41-48). Cells were cultured in complete medium comprising DMEM, 10% FBS, 2 mM L-glutamine, 1 mM D-glucose, 0.1% MEM NEAA (Merck), and 1% ABAM. Adult primary human chondrocytes were isolated from waste nasoseptal surgical tissue collected with informed consent from healthy donors undergoing septorhinoplasty at Singleton and Morriston Hospitals, Swansea, UK, with approval from the Swansea Bay University Health Board (IRAS ID 99202).
Primary bovine endometrial epithelial cells were isolated from normal uteri, which were collected from cattle at the slaughterhouse, as described previously (Amos MR, et al. 2014 Biol Reprod 90, 54). Cells were cultured in complete medium comprising RPMI 1640, 10% FBS, 2 mM L-glutamine, and 1% ABAM. Bovine endometrial epithelial cells were isolated from female genital tracts collected from cattle after they were slaughtered during the normal work of a commercial slaughterhouse, with approval from the United Kingdom Department for Environment, Food and Rural Affairs under the animal by-products regulation (EC) No. 1069/2009 (registration number U1268379/ABP/OTHER).

Authentication

Cell lines were newly purchased, or isolated as primary cells, and had expected morphology and growth characteristics.

Mycoplasma contamination

Cells were tested Mycoplasma negative.

Commonly misidentified lines
(See ICLAC register)

Name any commonly misidentified cell lines used in the study and provide a rationale for their use.
